# Augmenting the Anti‐Leukemic Activity of the BCL‐2 Inhibitor Venetoclax Through Its Transformation Into Polypharmacologic Dual BCL‐2/HDAC1 and Dual BCL‐2/HDAC6 Inhibitors

**DOI:** 10.1002/ddr.70084

**Published:** 2025-05-15

**Authors:** Alexandria M. Chan, Christian Eberly, Brandon Drennen, Christopher C. Goodis, Zoe Wuyts, Curt I. Civin, Steven Fletcher

**Affiliations:** ^1^ Department of Pharmaceutical Sciences University of Maryland School of Pharmacy Baltimore Maryland USA; ^2^ Center for Stem Cell Biology & Regenerative Medicine, Department of Pediatrics University of Maryland School of Medicine Baltimore Maryland USA; ^3^ School of Chemistry Cardiff University Main Building Cardiff UK; ^4^ Center for Stem Cell Biology & Regenerative Medicine, Departments of Pediatrics and Pharmacology, Physiology & Drug Development University of Maryland School of Medicine Baltimore Maryland USA; ^5^ University of Maryland Greenebaum Cancer Center Baltimore Maryland USA

**Keywords:** AML, BCL‐2, cancer, HDAC, venetoclax

## Abstract

Motivated by the anti‐leukemic synergy between histone deacetylase (HDAC) inhibitors and the FDA‐approved BCL‐2 inhibitor venetoclax, coupled with our interests in polypharmacology, we sought to bolster the anti‐leukemic efficacy of the clinical drug by grafting HDAC1‐selective or HDAC6‐selective inhibitor motifs onto a solvent‐accessible domain of venetoclax. We discovered multiple polypharmacological agents that both retained the potent BCL‐2 inhibitory activity of venetoclax and effectively inhibited either HDAC1 or HDAC6 with excellent (up to 80‐fold) selectivities for the desired HDAC isoform. In addition, relative to parental venetoclax, two of our lead compounds, **BD‐4‐213** and **AMC‐4‐154**, exhibited superior activities against the acute myeloid leukemia cell line MV4;11 and an MV4;11 cell line engineered to overexpress BCL‐2. Annexin‐V assay results confirmed an on‐target mechanism of apoptosis for these novel chimeric molecules. Efforts to further boost the HDAC1 or HDAC6 binding affinities and/or selectivities proved unsuccessful due to synthetic chemistry challenges and solubility problems, which may underscore the difficulties of polypharmacology approaches involving a large inhibitor, such as venetoclax.

## 1 Introduction

Acquired resistance to cell death is a hallmark of cancer, and one of the more prominent molecular pathways hijacked in the development and progression of cancer is the intrinsic pathway of apoptosis, governed by the BCL‐2 family of proteins (Adams and Cory 2018; Czabotar et al. 2014). Within the BCL‐2 family are pro‐survival proteins, which include BCL‐2 itself, BCL‐xL, MCL‐1, BFL‐1, and BCL‐w, and pro‐death counterparts that span two sub‐families: the effector proteins, such as BAK and BAX, and the BH3‐only proteins, including BIM, NOXA, and PUMA (Adams and Cory 2018). One BCL‐2 family member may neutralize an opposing family member's function via a protein–protein interaction mediated by an α‐helical BH3 “death” domain on the pro‐death protein that docks into the BH3‐binding groove on the surface of a given pro‐survival protein to functionally sequester the former. Venetoclax, a *bona fide* synthetic BH3 mimetic that is a selective inhibitor of the BCL‐2 protein (Souers et al. 2013), is approved for use in several hematologic malignancies, including acute myeloid leukemia (AML) and chronic lymphocytic leukemia (CLL), either as a monotherapy or in drug combinations (Gibson et al. 2021; Lasica and Anderson 2021). However, current venetoclax‐based therapies are not curative owing to the development of resistance (Tambe et al. 2024). Molecular mechanisms of resistance include overexpression of sister pro‐survival proteins, most commonly MCL‐1 (and also BCL‐xL) (Zhang et al. 2022), that seize prodeath BCL‐2 counterparts (Adams and Cory 2018). The last couple of decades have experienced a plethora of research programs dedicated to the discovery of inhibitors of the pro‐survival BCL‐2 proteins, especially BCL‐2 itself, BCL‐xL and MCL‐1, from a wide range of research groups, including ours (Cao et al. 2013; Chen et al. 2016, 2022, 2023; Conlon et al. 2020; Drennen et al. 2016; Fletcher 2019; Friberg et al. 2013; Lanning et al. 2015, 2016; Lee et al. 2019; Pelz et al. 2016; Souers et al. 2013; Yap et al. 2017).

The family of histone deacetylases (HDACs) is another chief target for the development of anticancer therapeutics. Through catalyzing the deacetylation of *N*‐terminal lysine residues of histones (incidentally, non‐histone substrates are continually being discovered), the HDACs are involved in the control of chromatin structural dynamics, and this biochemical reaction results in the compaction of chromatin and the repression of gene transcription (Ho et al. 2020). There are 18 HDAC isozymes divided into zinc‐dependent and nicotinamide adenine dinucleotide (NAD^+^)‐dependent enzymes; the Zn‐dependent HDACs are organized into four classes: Class I comprises HDAC1‐3 and HDAC8; Class IIa includes HDACs 4, 5, 7 and 9; Class IIb consists of HDACs 6 and 10; finally, HDAC11 is the only member of Class IV (Ho et al. 2020). The NAD^+^‐dependent HDACs, classically referred to as the sirtuins (sirt 1‐7), constitute Class III. While three HDAC inhibitors (HDACi's) have been approved by the FDA for various hematologic malignancies, they are associated with several side effects; it is speculated that this is due, at least in part, to their pan‐HDAC inhibitory activities (Ho et al. 2020). Accordingly, there is intense interest to develop isoform‐selective HDACi's, notably of HDAC1 and HDAC6 (and HDAC8) (Deng et al. 2023; Ho et al. 2020) HDAC1 is overexpressed in a wide range of solid tumors as well as in several leukemias and lymphomas, including acute lymphoblastic leukemia (ALL), CLL and AML (Deng et al. 2023). HDAC6 is also being targeted towards treatments for these leukemias and lymphomas, together with multiple myeloma and neurological diseases (Losson et al. 2020). (Last, although outside the scope of the present research, HDAC8 is emerging as a target for neuroblastoma (Deng et al. 2023; Zhao et al. 2024).)

Given the heterogeneity of cancers, the prescribed therapeutic regimens are often drug cocktails, or polypharmacy. Venetoclax and its relatives are currently being investigated in combination with HDACi's for multiple different cancers. For example, the pan‐HDACi SAHA (vorinostat) potentiated the therapeutic efficacy of the dual BCL‐2/BCL‐xL inhibitor ABT‐737 (a predecessor to venetoclax) to human leukemia and myeloma cells, partially through the upregulation of the pro‐death BCL‐2 protein BIM (Chen et al. 2009). In addition, synergy was observed between SAHA and venetoclax in cutaneous T‐cell lymphoma patient‐derived cells (Cyrenne et al. 2017), as well as between the HDAC1/2/3‐selective inhibitor chidamide and venetoclax in AML cells (Gangping, et al. 2021). Furthermore, the combination of venetoclax with the Class I (HDAC1‐3‐targeting) inhibitor chidamide and azacitidine demonstrated promising efficacy and an acceptable safety profile in patients with relapsed/refractory (R/R) AML (Zha et al. 2023).

The field of polypharmacology has recently emerged, wherein single drugs are developed to inhibit multiple protein targets, either through (a) the merging of two (or more) pharmacophoric ligands into a single ligand, or (b) the linking of two (or more) pharmacophoric ligands to arrive at a bivalent (or polyvalent) inhibitor (Anighoro et al. 2014; Proschak et al. 2019). In fact, most of the FDA‐approved receptor tyrosine kinase (RTK) drugs serendipitously inhibit multiple RTKs through a single, “promiscuous” ligand (Pottier et al. 2020), and thus fall into the former class of polypharmacology. Although advantages of the latter (linking) type of polypharmacology over the corresponding polypharmacy/drug cocktail therapeutic regimen are yet to be observed in the clinic, increased patient compliance is a potential practical benefit through the reduction in the number of medications the patient would be prescribed, as well as enhanced synergy through the guaranteed co‐delivery of both ligands to the site of action, among others (Anighoro et al. 2014; Proschak et al. 2019). Polypharmacologic HDAC inhibitors, in particular HDAC6‐targeting agents, were recently reviewed (Chan and Fletcher 2021). Previously, Zhou and co‐workers transformed venetoclax into several dual BCL‐2/HDAC inhibitors by combining venetoclax with the pan‐HDACi SAHA (Zhou et al. 2019). Surprisingly, these dual inhibitors yielded enhanced selectivities for HDAC6 over HDAC1, relative to the parent drug SAHA (from about threefold to 30‐fold), while inhibitory activities to the other HDAC isozymes were not provided. As was noted earlier, isoform‐selective HDACi's are fervently being sought. By extension, in the context of dual BCL‐2/HDAC inhibitors, we believe it is of importance to investigate if HDAC‐selective inhibitor motifs may be grafted onto a BCL‐2 inhibitor such as venetoclax without any erosion in isoform selectivity to yield BCL‐2/HDAC dual inhibitors that retain specificity for particular HDAC isoforms.

Previously, we prepared dual HDAC/proteasome inhibitors by grafting HDAC1‐ or HDAC6‐selective inhibitor motifs onto a proteasome inhibitor, and observed that HDAC isoform selectivities associated with the parental HDAC inhibitors were preserved in the resulting polypharmacologic inhibitors (Chan et al. 2024). Herein, we explored a similar strategy to construct chimeric HDAC/BCL‐2 dual inhibitors, comprising HDAC inhibitor motifs coupled to venetoclax. In particular, given the association of HDAC1 and HDAC6 with the progression of AML and CLL – two hematologic cancers for which venetoclax has already received FDA approval – we elected to focus on achieving HDAC1 and HDAC6 selectivity in our dual BCL‐2/HDAC inhibitors.

## 2 Methods

### 2.1 General

All reactions were performed in oven‐dried glassware under an inert (N_2_) atmosphere, unless otherwise stated. Reactions were monitored by thin layer chromatography (TLC), visualizing with UV_254_ or KMnO_4_ stain. Anhydrous solvents were used as supplied without further purification. ^1^H and ^13^C NMR spectra were recorded on a Varian 400 MHz NMR spectrometer at 25°C. Chemical shifts are reported in parts per million (ppm) and are referenced to residual non‐deuterated solvent peak (CHCl_3_: δ_H_ 7.26, δ_C_ 77.2; DMSO: δ_H_ 2.50, δ_C_ 39.5).

#### 2.1.1 General Procedure A: HATU‐Mediated Coupling

To a stirring solution of the carboxylic acid (1.0 eq) in DMF (0.2 M), DIPEA (1.1 eq) and HATU (1.1 eq) were added followed by the amine (1.0 eq) and additional 1.0 eq DIPEA per HCl salt. The reaction was stirred at room temperature for 16 h.

#### 2.1.2 General Procedure B: Boc‐Deprotection of Amines

To a stirring solution of the Boc‐protected amine (1.0 eq) in CH_2_Cl_2_ (0.1 M), trifluoroacetic acid (TFA) (0.1 M) was added. The reaction mixture was stirred at room temperature for 1 h. Upon completion, the reaction mixture was concentrated to dryness and azeotroped with CHCl_3_ and methanol (2x), then CHCl_3_.

#### 2.1.3 General Procedure C: EDCI‐Condensation With Venetoclax Acid **7** (Ku et al. 2019)

To a stirring solution of the amine (1.0 eq) in CH_2_Cl_2_ (0.1 M), 2‐((1H‐pyrrolo[2,3‐b]pyridine‐5‐yl)oxy)‐4‐(4‐((4′‐chloro‐5,5‐dimethyl‐3,4,5,6‐tetrahydro‐[1,1′‐biphenyl]‐2‐yl)methyl)piperzin‐1‐yl)benzoic acid (**7**; 1.0 eq) was added, followed by EDCI (1.3 eq), DMAP (1.0 eq) and DIPEA (2.0 eq). The reaction mixture was stirred at room temperature for 16 h. Upon completion, *N,N*‐dimethylethylenediamine was added and the reaction mixture was stirred for 1 h. The reaction mixture was diluted in CH_2_Cl_2_ and washed with 10% acetic acid in water and 5% saturated sodium bicarbonate in water, then dried over sodium sulfate, filtered and concentrated to dryness.

#### 2.1.4 General Procedure D: Saponification of Esters to Carboxylic Acids

The ester (1.0 eq) was dissolved in THF/MeOH/H_2_O, 3:1:1 (0.1 M), and lithium hydroxide monohydrate (4.0 eq) was added. The reaction mixture was stirred at room temperature for 16 h. Upon completion, the reaction mixture was diluted with ethyl acetate and the lithium hydroxide was neutralized with stoichiometric 1 M HCl. The reaction was partitioned between ethyl acetate and saturated sodium phosphate monobasic buffer. The product was extracted from the aqueous into ethyl acetate (3x). The organic layers were combined and dried over sodium sulfate, filtered and then concentrated to dryness.

#### 2.1.5 General Procedure E: Nucleophilic Aromatic Substitution (S_N_Ar) Reactions With Aryl Fluoride **5**

Compound **5** (1.0 eq) was dissolved in DMF (0.1 M). The amine (1.1 eq) was added followed by potassium carbonate (3.0 eq) and the reaction mixture was stirred at room temperature for 16 h. Upon completion, the reaction mixture was diluted in ethyl acetate and partitioned with water. The product was extracted into ethyl acetate (3x). The organic layers were combined, washed with water (3x) and saturated sodium chloride, then dried over sodium sulfate, filtered and concentrated to dryness.

#### 2.1.6 General Procedure F: Palladium Catalyzed Reduction of Nitro Groups

To a stirring solution of the starting material (1.0 eq) in methanol (0.1 M) was added Pd/C (10% wt). Hydrogen gas was bubbled through the solution for 10 min. Then the reaction mixture was stirred at room temperature for 16 h under a balloon of H_2_. Upon completion, the reaction mixture was filtered through celite, rinsing with methanol and concentrated to dryness.

#### 2.1.7 General Procedure G: THP‐Deprotection of Hydroxamic Acids

The THP‐protected hydroxamic acid (1.0 eq) was dissolved in methanol (0.1 M). Trifluoroacetic acid (5.0 eq) was added and the reaction mixture was stirred at room temperature for 2 h. Upon completion, the reaction mixture was concentrated to dryness and azeotroped with CHCl_3_/methanol (2x) then CHCl_3_.

*4‐Fluoro‐3‐nitrobenzenesulfonamide (**5**)*: (Ku et al. 2019) 2‐fluoronitrobenzene (35.44 mmol) was dissolved in chlorosulfonic acid (11 mL) and refluxed for 16 h. The reaction was cooled to room temperature, and then slowly added to a solution of 30 mL ammonium hydroxide in 110 mL of isopropanol at −40°C. The product was re‐acidified and collected by vacuum filtration, washing with water, and dried in a vacuum oven for 16 h, yielding 4‐fluoro‐3‐nitrobenzenesulfonamide (**5**) as a yellow solid with no further purification required (yield = 93%): ^1^H NMR (400 MHz, *d*_*6*_‐DMSO) δ8.56 (1H, dd, *J* = 2.4 Hz), 8.22 (1H, m), 7.85 (1H, dd, *J* = 8.8 Hz), 7.76 (2H, s).

*Methyl 4‐(((2‐nitro‐4‐sulfamoylphenyl)amino)methyl)benzoate (**6**):* 4‐(aminomethyl) benzoic acid (**4**; 16.5 mmol, 1.0 eq) was dissolved in methanol (0.1 M) and cooled to 0°C. Thionyl chloride (41.3 mmol, 2.5 eq) was added dropwise and the reaction mixture was refluxed at 60°C for 16 h. Upon completion, the reaction mixture was concentrated to dryness. The crude product was vigorously stirred in ether for 1 h before collection by vacuum filtration, resulting in methyl 4‐(aminomethyl)benzoate as its hydrochloride salt (white solid, yield = 89%). 4‐Fluoro‐3‐nitrobenzenesulfonamide (**5**; 6.0 mmol, 1.0 eq) was dissolved in DMF (0.1 M), then methyl 4‐(aminomethyl)benzoate. HCl (6.6 mmol, 1.1 eq) was added, followed by potassium carbonate (18.0 mmol, 3.0 eq), and the reaction mixture was stirred at room temperature for 16 h. Upon completion, the product was precipitated in cold water and collected by vacuum filtration, washing with cold water, drying on the filter and then in a vacuum oven, resulting in methyl 4‐(((2‐nitro‐4‐sulfamoylphenyl)amino)methyl)benzoate (**6**) as a yellow solid that needed no further purification (yield = 99%): ^1^H NMR (400 MHz, *d*_*6*_‐DMSO) δ 9.10 (1H, s), 8.48 (1H, s), 7.92 (2H, d, *J* = 7.6 Hz), 7.71 (1H, d, *J* = 9.6 Hz), 7.48 (2H, d, *J* = 8 Hz), 6.97 (1H, d, *J* = 9.6 Hz), 4.78 (2H, d, *J* = 5.2 Hz), 3.81 (3H, s).

*4‐(((4‐(N‐(2‐((1H‐pyrrolo[2,3‐b]pyridin‐5‐yl)oxy)‐4‐(4‐((4′‐chloro‐5,5‐dimethyl‐3,4,5,6‐tetrahydro‐[1,1′‐biphenyl]‐2‐yl)methyl)piperazin‐1‐yl)benzoyl)sulfamoyl)‐2‐nitrophenyl)amino)methyl)benzoic acid (**8**):* Sulfonamide **6** (1.0 mmol) was reacted with carboxylic acid **7** according to *General Procedure C*. The product was isolated via flash column chromatography over silica gel eluting with a mixture of CH_2_Cl_2_/MeOH/H_2_O, 79:9:1 resulting in methyl 4‐(((4‐(*N*‐(2‐((1*H*‐pyrrolo[2,3‐*b*]pyridin‐5‐yl)oxy)‐4‐(4‐((4′‐chloro‐5,5‐dimethyl‐3,4,5,6‐tetrahydro‐[1,1′‐biphenyl]‐2‐yl)methyl)piperazin‐1‐yl)benzoyl)sulfamoyl)‐2‐nitrophenyl)amino)methyl)benzoate as a yellow solid (yield = 93%). Subsequently, this product (0.816 mmol) was saponified according to *General Procedure D*, resulting in 4‐(((4‐(*N*‐(2‐((1*H*‐pyrrolo[2,3‐*b*]pyridin‐5‐yl)oxy)‐4‐(4‐((4′‐chloro‐5,5‐dimethyl‐3,4,5,6‐tetrahydro‐[1,1′‐biphenyl]‐2‐yl)methyl)piperazin‐1‐yl)benzoyl)sulfamoyl)‐2‐nitrophenyl)amino)methyl)benzoic acid (**8**) as a yellow solid that needed no further purification (yield = 99%): ^1^H NMR (400 MHz, *d*_*6*_‐DMSO) δ12.64 (1H, s), 11.70 (1H, s), 11.37 (1H, s), 9.16 (1H, s), 8.57 (1H, s), 8.01 (1H, s), 7.88 (2H, d, *J* = 8.0 Hz), 7.69 (1H, d, *J* = 9.6 Hz), 7.50 (2H, s), 7.44 (2H, d, *J* = 6.8 Hz), 7.32 (2H, d, *J* = 7.6 Hz), 7.01 (2H, d, *J* = 8.0 Hz), 6.81 (1H, d, 9.2 Hz), 6.64 (1H, d, *J* = 8.8 Hz), 6.36 (1H, app. t), 6.15 (1H, s), 4.72 (2H, d, *J* = 5.6 Hz), 3.04 (4H, s), 2.73 (2H, s), 2.15 (6H, m), 1.93 (2H, s), 1.36 (2H, m), 0.90 (6H, s).

*2‐((1H‐pyrrolo[2,3‐b]pyridin‐5‐yl)oxy)‐4‐(4‐((4′‐chloro‐5,5‐dimethyl‐3,4,5,6‐tetrahydro‐[1,1′‐biphenyl]‐2‐yl)methyl)piperazin‐1‐yl)‐N‐((4‐((4‐(hydroxycarbamoyl)benzyl)amino)‐3‐nitrophenyl)sulfonyl)benzamide (**SF‐8‐038** (**10**)):* Compound **8** (0.592 mmol) was reacted with *O*‐THP‐hydroxylamine (0.651 mmol) according to *General Procedure A*. Upon completion, the reaction mixture was diluted in ethyl acetate and partitioned with water. The product was extracted into ethyl acetate (3x). The organic layers were combined and washed with water (3x) and saturated sodium chloride, then dried over sodium sulfate, filtered and concentrated to dryness, yielding crude 2‐((1*H*‐pyrrolo[2,3‐b]pyridin‐5‐yl)oxy)‐4‐(4‐((4′‐chloro‐5,5‐dimethyl‐3,4,5,6‐tetrahydro‐[1,1′‐biphenyl]‐2‐yl)methyl)piperazin‐1‐yl)‐N‐((3‐nitro‐4‐((4‐(((tetrahydro‐2*H*‐pyran‐2‐yl)oxy)carbamoyl)benzyl)amino)phenyl)sulfonyl)benzamide that was used without further purification. Crude 2‐((1*H*‐pyrrolo[2,3‐b]pyridin‐5‐yl)oxy)‐4‐(4‐((4′‐chloro‐5,5‐dimethyl‐3,4,5,6‐tetrahydro‐[1,1′‐biphenyl]‐2‐yl)methyl)piperazin‐1‐yl)‐N‐((3‐nitro‐4‐((4‐(((tetrahydro‐2*H*‐pyran‐2‐yl)oxy)carbamoyl)benzyl)amino)phenyl)sulfonyl)benzamide (0.428 mmol) was reacted according to *General Procedure G*. The product was isolated via flash column chromatography over silica gel eluting with a mixture of CH_2_Cl_2_/MeOH/H_2_O, 79:9:1, yielding 2‐((1H‐pyrrolo[2,3‐b]pyridin‐5‐yl)oxy)‐4‐(4‐((4′‐chloro‐5,5‐dimethyl‐3,4,5,6‐tetrahydro‐[1,1′‐biphenyl]‐2‐yl)methyl)piperazin‐1‐yl)‐N‐((4‐((4‐(hydroxycarbamoyl)benzyl)amino)‐3‐nitrophenyl)sulfonyl)benzamide. (**SF‐8‐038** [**10**]) as a yellow solid (yield = 66%): ^1^H NMR (400 MHz, *d*_*6*_‐DMSO) δ11.68 (1H, s), 11.14 (1H, s), 9.12 (1H, s), 8.99 (1H, s), 58.57 (1H, s), 8.02 (1H, s), 7.69 (3H, m), 7.45 (5H, m), 7.33 (2H, d, *J* = 6.4 Hz), 7.02 (2H, d, *J* = 6.4 Hz), 6.84 (1H, d, *J* = 8.4 Hz), 6.65 (1H, d, *J* = 7.2 Hz), 6.37 (1H, s), 6.16 (1H, s), 4.69 (2H, s), 3.05 (4H, s), 2.74 (2H, s), 2.15 (6H, m), 1.94 (3H, m), 1.37 (2H, app. t), 0.91 (6H, s); ^13^C NMR (500 MHz, *d*_*6*_‐DMSO) δ164.67, 164.44, 158.44, 155.12, 147.15, 146.03, 142.54, 141.59, 135.88, 134.53, 132.76, 131.47, 130.74, 130.61 (2), 128.70 (2), 128.20, 127.87 (2), 127.44 (2), 120.39, 118.47, 115.66, 109.38, 103.01, 100.59, 60.21, 52.60 (2), 47.09 (2), 46.92, 46.22, 35.41, 29.46, 28.51 (2), 25.76.; HRMS (ESI) m/z [M + H]+ calcd for C_47_H_47_ClN_8_O_8_S = 919.2999; found = 919.2995.

*2‐((1H‐pyrrolo[2,3‐b]pyridin‐5‐yl)oxy)‐N‐((4‐((4‐((2‐aminophenyl)carbamoyl)benzyl)amino)‐3‐nitrophenyl)sulfonyl)‐4‐(4‐((4′‐chloro‐5,5‐dimethyl‐3,4,5,6‐tetrahydro‐[1,1′‐biphenyl]‐2‐yl)methyl)piperazin‐1‐yl)benzamide (**BD‐4‐213** (**12**)):* To a stirring solution of *O‐*phenylenediamine (13.87 mmol, 1.0 eq) in anhydrous THF (100 mL), triethylamine (27.74 mmol, 2.0 eq) and Boc_2_O (13.87 mmol, 1.0 eq) were added at 0°C. The reaction mixture was stirred at room temperature for 16 h. Upon completion, the reaction mixture was concentrated to remove volatiles, diluted in ethyl acetate, and partitioned with saturated sodium chloride. The organic layer was dried over sodium sulfate and concentrated to dryness, yielding *tert*‐butyl (2‐aminophenyl)carbamate (**11**) that required no further purification, and spectral data were consistent with published data: (Lameijer et al. 2017) (tan solid, yield = 90%). Subsequently, **8** was coupled to **11** on a 2.9 mmol scale, according to *General Procedure A*. The product was isolated via flash column chromatography over silica gel using an eluent of CH_2_Cl_2_/MeOH/H_2_O, 179:9:1, resulting in *ter*t‐butyl (2‐(4‐(((4‐(*N*‐(2‐((1H‐pyrrolo[2,3‐b]pyridin‐5‐yl)oxy)‐4‐(4‐((4′‐chloro‐5,5‐dimethyl‐3,4,5,6‐tetrahydro‐[1,1′‐biphenyl]‐2‐yl)methyl)piperazin‐1‐yl)benzoyl)sulfamoyl)‐2‐nitrophenyl)amino)methyl)benzamido)phenyl)carbamate as a yellow solid (yield = 68%), which was subsequently deprotected on a 0.0549 mmol according to *General Procedure B*. The product was isolated via flash column chromatography over silica gel with an eluent of CH_2_Cl_2_/MeOH/H_2_O, 179:9:1, yielding 2‐((1*H*‐pyrrolo[2,3‐b]pyridin‐5‐yl)oxy)‐*N*‐((4‐((4‐((2‐aminophenyl)carbamoyl)benzyl)amino)‐3‐nitrophenyl)sulfonyl)‐4‐(4‐((4′‐chloro‐5,5‐dimethyl‐3,4,5,6‐tetrahydro‐[1,1′‐biphenyl]‐2‐yl)methyl)piperazin‐1‐yl)benzamide (**BD‐4‐213** [**12**]) as a yellow solid (yield = 60%): ^1^H NMR (400 MHz, *d*_*6*_‐DMSO) δ 11.65 (1H, s), 9.58 (1H, s), 9.11 (1H, s), 8.59 (1H, s), 7.99 (1H, s), 7.92 (2H, d, *J* = 7.6 Hz), 7.67 (1H, s), 7.47 (5H, d, *J* = 7.2 Hz), 7.33 (2H, s, *J* = 8.8 Hz), 7.14 (1H, d, *J* = 7.6 Hz), 7.03 (2H, d, *J* = 8.8 Hz), 6.94 (1H, t, *J* = 7.8 Hz), 6.75 (1H, d, *J* = 8 Hz), 6.64 (1H, d, *J* = 9.2 Hz), 6.57 (1H, t, *J* = 7.4 Hz), 6.35 (1H, s), 6.17 (1H, s), 4.88 (2H, s), 4.72 (2H, s), 3.30 (2H, s), 3.03 (4H, s), 2.72 (2H, s), 2.15 (6H, s), 1.94 (2H, s), 0.91 (6H, s); ^13^C NMR (500 MHz, *d*_*6*_‐DMSO) δ 171.17, 165.73, 158.00, 147.80, 143.71, 142.64, 142.01, 135.76, 134.86, 134.63, 134.29, 132.74, 131.43, 130.63 (2), 129.49, 129.31, 128.77, 128.68, 128.14, 127.36, 127.24, 127.04, 123.91, 120.34, 117.92, 116.79, 116.67, 115.39, 109.49, 108.25, 100.50, 64.53, 60.31, 52.73, 47.41, 46.93, 46.22, 36.41, 35.45, 31.38, 29.49 (2), 28.53 (2), 25.79; HRMS (ESI) m/z [M + H]+ calcd for C_53_H_52_ClN_9_O_7_S = 994.3472; found = 994.3483.

*2‐((1H‐pyrrolo[2,3‐b]pyridin‐5‐yl)oxy)‐4‐(4‐((4′‐chloro‐5,5‐dimethyl‐3,4,5,6‐tetrahydro‐[1,1′‐biphenyl]‐2‐yl)methyl)piperazin‐1‐yl)‐N‐((4‐(((1‐(4‐(hydroxycarbamoyl)phenyl)piperidin‐4‐yl)methyl)amino)‐3‐nitrophenyl)sulfonyl)benzamide (**AMC‐4‐154** (**18**)):* To a solution of 4‐fluorobenzonitirle (0.413 mmol, 1.0 eq) in DMSO (0.1 M), 4‐(Boc‐aminomethyl)piperidine (0/413 mmol, 1.0 eq) and potassium carbonate (0.826 mmol, 2.0 eq) were added and the reaction mixture was stirred at 90°C for 16 h. Upon completion, the reaction mixture was cooled to room temperature, diluted with ethyl acetate, and partitioned with water. The product was extracted into additional ethyl acetate (3x). The organic layers were combined and washed with 1 M HCl, water (3x), and saturated sodium chloride, then dried over sodium sulfate and concentrated to dryness, resulting in *tert*‐butyl ((1‐(4‐cyanophenyl)piperidin‐4‐yl)methyl)carbamate without further purification (white solid, yield = 50%) (Nikiforov et al. 2016). *Tert*‐butyl ((1‐(4‐cyanophenyl)piperidin‐4‐yl)methyl)carbamate (0.159 mmol) was dissolved in 25% (w/w) aqueous potassium hydroxide:ethanol (0.2 M) and stirred at 80°C for 16 h. Upon completion, the reaction mixture was cooled to room temperature and dissolved in ethyl acetate. The aqueous layer was acidified with 1 M HCl and the product was extracted into additional ethyl acetate (3x). The organic layers were combined, dried over sodium sulfate and concentrated to dryness, resulting in 4‐(4‐(((tert‐butoxycarbonyl)amino)methyl)piperidin‐1‐yl)benzoic acid (**15**) that was used without additional purification (tan solid, yield = 99%). 4‐(4‐(((*tert*‐butoxycarbonyl)amino)methyl)piperidin‐1‐yl)benzoic acid (**15**; 0.075 mmol, 1.0 eq) was dissolved in DMF (0.1 M). Potassium carbonate (0.15 mmol, 1.5 eq) was added, followed by iodomethane (0.150 mmol, 2.0 eq) and the reaction mixture was stirred at room temperature for 16 h. Upon completion, the reaction mixture was dissolved in ethyl acetate and partitioned with water. The product was extracted into additional ethyl acetate (3x). The organic layers were combined, washed with saturated sodium bicarbonate, water (3x), and saturated sodium chloride, then dried over sodium sulfate, filtered and concentrated to dryness, resulting in methyl 4‐(4‐(((*tert*‐butoxycarbonyl)amino)methyl)piperidin‐1‐yl)benzoate (**15b**) as a tan solid that needed no further purification (yield = 91%): ^1^H NMR (400 MHz, CDCl_3_) δ 7.92 (2H, d, *J* = 8.8 Hz), 6.88 (2H, d, *J* = 7.6 Hz), 4.25 (1H, s), 3.92–3.88 (5H, m), 3.08 (2H, s), 2.85 (2H, t, *J* = 12.0 Hz), 1.82 (2H, d, *J* = 12.4 Hz), 1.47 (10H, s), 1.38–1.28 (2H, m).

Methyl 4‐(4‐(((tert‐butoxycarbonyl)amino)methyl)piperidin‐1‐yl)benzoate (**15b**; 0. 956 mmol) was reacted according to *General Procedure B*, resulting in methyl 4‐(4‐(aminomethyl)piperidin‐1‐yl)benzoate trifluoroacetate (**16**), which was used without further purification (tan solid, yield = 99%). Methyl 4‐(4‐(aminomethyl)piperidin‐1‐yl)benzoate trifluoroacetate (**16**; 0.956 mmol) was reacted according to *General Procedure E*. The product was isolated via flash column chromatography over silica gel, eluting with a mixture of CH_2_Cl_2_/MeOH/NH_4_OH, 92:7:1, furnishing methyl 4‐(4‐(((2‐nitro‐4‐sulfamoylphenyl)amino)methyl)piperidin‐1‐yl)benzoate as a yellow solid (yield = 74%), which was then reacted according to *General Procedure C* on a 0.350 mmol scale. The product was isolated via flash column chromatography over silica gel, eluting with a mixture of CH_2_Cl_2_/MeOH/NH_4_OH, 21:7:1, resulting in methyl 4‐(4‐(((4‐(*N*‐(2‐((1*H*‐pyrrolo[2,3‐*b*]pyridin‐5‐yl)oxy)‐4‐(4‐((4′‐chloro‐5,5‐dimethyl‐3,4,5,6‐tetrahydro‐[1,1′‐biphenyl]‐2‐yl)methyl)piperazin‐1‐yl)benzoyl)sulfamoyl)‐2‐nitrophenyl)amino)methyl)piperidin‐1‐yl)benzoate (**17**) as a yellow solid (yield = 81%): (400 MHz, *d*_*6*_‐DMSO) δ 11.63 (1H, s), 8.58 (1H, s), 8.51 (1H, s), 8.00 (1H, s), 7.75 (2H, d, *J* = 8.8 Hz), 7.50‐7.46 (2H, m), 7.33 (2H, d, *J* = 8.4 Hz), 7.02 (2H, d, *J* = 8.0 Hz), 6.95 (2H, d, *J* = 8.4 Hz), 6.65 (1H, d, *J* = 9.6 Hz), 6.35 (1H, s), 6.18 (1H, s), 3.93 (2H, d, *J* = 12.8 Hz), 3.75 (2H, s), 3.32 (4H, s), 3.04 (4H, s), 2.81 (2H, t, *J* = 12.2 Hz), 2.71 (2H, s), 2.16 (6H, s), 1.93 (2H, s), 1.76 (2H, d, *J* = 12.8 Hz), 1.37 (2H, t, *J* = 5.6 Hz), 1.26 (2H, q, *J* = 10.9 Hz), 0.91 (6H, s).

Methyl 4‐(4‐(((4‐(*N*‐(2‐((1*H*‐pyrrolo[2,3‐*b*]pyridin‐5‐yl)oxy)‐4‐(4‐((4′‐chloro‐5,5‐dimethyl‐3,4,5,6‐tetrahydro‐[1,1′‐biphenyl]‐2‐yl)methyl)piperazin‐1‐yl)benzoyl)sulfamoyl)‐2‐nitrophenyl)amino)methyl)piperidin‐1‐yl)benzoate (**17**; 0.200 mmol) was reacted according to *General Procedure D*, resulting in 4‐(4‐(((4‐(*N*‐(2‐((1*H*‐pyrrolo[2,3‐*b*]pyridin‐5‐yl)oxy)‐4‐(4‐((4′‐chloro‐5,5‐dimethyl‐3,4,5,6‐tetrahydro‐[1,1′‐biphenyl]‐2‐yl)methyl)piperazin‐1‐yl)benzoyl)sulfamoyl)‐2‐nitrophenyl)amino)methyl)piperidin‐1‐yl)benzoic acid (**17b**) without further purification (yellow solid, yield = 74%). 4‐(4‐(((4‐(*N*‐(2‐((1*H*‐pyrrolo[2,3‐*b*]pyridin‐5‐yl)oxy)‐4‐(4‐((4′‐chloro‐5,5‐dimethyl‐3,4,5,6‐tetrahydro‐[1,1′‐biphenyl]‐2‐yl)methyl)piperazin‐1‐yl)benzoyl)sulfamoyl)‐2‐nitrophenyl)amino)methyl)piperidin‐1‐yl)benzoic acid (**17b**; 0.294 mmol) was reacted according with *O*‐THP‐hydroxylamine (0.323 mmol) according to *General Procedure A*. Upon completion, the reaction mixture was dissolved in ethyl acetate and partitioned with water. The product was extracted from the aqueous with additional ethyl acetate (3x). The organic layers were combined and washed with water (3x) and saturated sodium chloride, then dried over sodium sulfate, filtered and concentrated to dryness. The product was isolated via flash column chromatography over silica gel, eluting with a mixture of CH_2_Cl_2_/MeOH/NH_4_OH, 21:7:1, resulting in 2‐((1*H*‐pyrrolo[2,3‐*b*]pyridin‐5‐yl)oxy)‐4‐(4‐((4′‐chloro‐5,5‐dimethyl‐3,4,5,6‐tetrahydro‐[1,1′‐biphenyl]‐2‐yl)methyl)piperazin‐1‐yl)‐*N*‐((3‐nitro‐4‐(((1‐(4‐(((tetrahydro‐2*H*‐pyran‐2‐yl)oxy)carbamoyl)phenyl)piperidin‐4‐yl)methyl)amino)phenyl)sulfonyl)benzamide (**17c**; yellow solid, yield = 43%). 2‐((1*H*‐pyrrolo[2,3‐*b*]pyridin‐5‐yl)oxy)‐4‐(4‐((4′‐chloro‐5,5‐dimethyl‐3,4,5,6‐tetrahydro‐[1,1′‐biphenyl]‐2‐yl)methyl)piperazin‐1‐yl)‐*N*‐((3‐nitro‐4‐(((1‐(4‐(((tetrahydro‐2*H*‐pyran‐2‐yl)oxy)carbamoyl)phenyl)piperidin‐4‐yl)methyl)amino)phenyl)sulfonyl)benzamide (**17c**; 0.127 mmol) was reacted according to *General Procedure G*. The crude product was dissolved in ethyl acetate and partitioned with water. The aqueous layer was basified with saturated sodium bicarbonate and extracted into additional ethyl acetate (2x). The organic layers were combined and dried over sodium sulfate then concentrated to dryness. The product was isolated via preparatory thin layer chromatography over silica gel, eluting with a mixture of CH_2_Cl_2_/MeOH/AcOH, 75:21:1, resulting in 2‐((1*H*‐pyrrolo[2,3‐*b*]pyridin‐5‐yl)oxy)‐4‐(4‐((4′‐chloro‐5,5‐dimethyl‐3,4,5,6‐tetrahydro‐[1,1′‐biphenyl]‐2‐yl)methyl)piperazin‐1‐yl)‐*N*‐((4‐(((1‐(4‐(hydroxycarbamoyl)phenyl)piperidin‐4‐yl)methyl)amino)‐3‐nitrophenyl)sulfonyl)benzamide. (**AMC‐4‐154** [**18**]) as a yellow solid (yield = 88%): ^1^H NMR (400 MHz, *d*_*6*_‐DMSO) δ 11.63 (1H, s), 10.89 (1H, s), 8.74 (1H, s), 8.64 (1H, s), 8.56 (1H, s), 8.03 (1H, s), 7.79 (1H, d, *J* = 8.8 Hz), 7.60 (2H, d, *J* = 8.4 Hz), 7.54 (1H, s), 7.48 (2H, d, *J* = 8.8 Hz), 7.33 (2H, d, *J* = 7.2 Hz), 7.13 (1H, d, *J* = 9.2 Hz), 7.02 (2H, d, *J* = 8.0 Hz), 6.91 (2H, d, *J* = 8.8 Hz), 6.67 (1H, d, *J* = 8.4 Hz), 6.38 (1H, s), 6.18 (1H, s), 3.86 (2H, d, *J* = 11.6 Hz), 3.08 (4H, m), 2.73 (3H, t, *J* = 12.4 Hz), 2.29 (3H, m), 2.13 (3H, m), 1.94 (2H, m(, 1.76 (2H, d, *J* = 11.6 Hz), 1.38 (2H, app. t), 1.27 (3H, m), 0.91 (6H, s); ^13^C NMR: (500 MHz, *d*_*6*_‐DMSO) δ 164.27, 158.47, 154.98, 147.98, 147.08, 146.07, 142.25, 135.88, 134.52, 132.72, 131.63, 130.56 (2), 130.18 (2), 128.79 (2), 128.38 (2), 125.32, 121.75, 120.41, 118.52, 115.68, 114.44 (2), 113.28, 109.42, 103.05, 100.59, 59.91, 52.31, 48.29, 47.87, 46.98, 46.57, 35.46, 35.30, 29.42, 29.37, 28.49 (2), 25.66; HRMS (ESI) m/z [M + H]+ calcd for C_52_H_56_ClN_9_O_8_S = 1002.3734; found = 1002.3746.

2‐((2‐((4‐(*N*‐(2‐((1*H*‐pyrrolo[2,3‐b]pyridin‐5‐yl)oxy)‐4‐(4‐((4′‐chloro‐5,5‐dimethyl‐3,4,5,6‐tetrahydro‐[1,1′‐biphenyl]‐2‐yl)methyl)piperazin‐1‐yl)benzoyl)sulfamoyl)‐2‐nitrophenyl)amino)ethyl)amino)‐N‐hydroxypyrimidine‐5‐carboxamide (**BD‐4‐208**): To a stirring solution of ethyl 2‐chloropyrimidine‐5‐carboxylate (5.36 mmol, 1.0 eq) in acetonitrile (0.1 M) was added ethylenediamine (53.6 mmol, 10 eq). The reaction was stirred at room temperature for 16 h. Upon completion, the reaction mixture was concentrated to remove volatiles, diluted with ethyl acetate, and partitioned with water. The product was extracted into additional ethyl acetate (3x). The organic layers were combined and washed with water and saturated sodium chloride, then dried over sodium sulfate, filtered and concentrated to dryness. No further purification was required, furnishing ethyl 2‐((2‐aminoethyl)amino)pyrimidine‐5‐carboxylate (**19**): (off‐yellow solid, 90%): ^1^H NMR (400 MHz, DMSO) δ = 8.70 (d, J = 18.8 Hz, 2H), 8.09 (s, 1H), 4.24 (q, J = 7.1 Hz, 2H), 4.01 (s, 2H), 3.48 (s, 1H), 3.35 (s, 1H), 3.10 (s, 1H), 2.72 (s, 1H), 1.27 (t, J = 7.2 Hz, 3H). Ethyl 2‐((2‐aminoethyl)amino)pyrimidine‐5‐carboxylate (1.375 mmol) was then reacted according to *General Procedure E*, delivering ethyl 2‐((2‐((2‐nitro‐4‐sulfamoylphenyl)amino)ethyl)amino)pyrimidine‐5‐carboxylate (**20**) as a yellow solid (yield = 95%): ^1^H NMR (400 MHz, *d*_6_‐DMSO) δ = 8.84 (s, 1H), 8.77–8.75 (m, 2H), 8.49 (s, 1H), 8.35 (s, 1H), 7.90 (d, J = 9.2 Hz, 1H), 7.41 (d, J = 9.6 Hz, 1H), 7.36 (s, 2H), 4.30 (q, J = 7.2 Hz, 2H), 3.65 (s, 4H), 1.32 (t, J = 7.0 Hz, 3H). Ethyl 2‐((2‐((2‐nitro‐4‐sulfamoylphenyl)amino)ethyl)amino)pyrimidine‐5‐carboxylate (**20**; 0.175 mmol) was reacted according to *General Procedure C*. The product was isolated via flash column chromatography over silica gel eluting with a mixture of CH_2_Cl_2_/MeOH/NH_4_OH, 75:21:1, resulting in ethyl 2‐((2‐((4‐(N‐(2‐((1H‐pyrrolo[2,3‐b]pyridin‐5‐yl)oxy)‐4‐(4‐((4′‐chloro‐5,5‐dimethyl‐3,4,5,6‐tetrahydro‐[1,1′‐biphenyl]‐2‐yl)methyl)piperazin‐1‐yl)benzoyl)sulfamoyl)‐2‐nitrophenyl)amino)ethyl)amino)pyrimidine‐5‐carboxylate (**21**) as a yellow solid (yield = 71%): ^1^H NMR (400 MHz, *d*_6_‐DMSO) δ = 11.69 (s, 1H), 8.84 (s, 1H), 8.78 (s, 1H), 8.74 (s, 1H), 8.35 (s, 1H), 8.05 (s, 1H), 7.85 (d, J = 9.2 Hz, 1H), 7.54–7.50 (m, 3H), 7.36 (d, J = 8.0 Hz, 2H), 7.19 (d, J = 9.6 Hz, 1H), 7.06 (d, J = 8.4 Hz, 2H), 6.69 (d, J = 8.4 Hz, 1H), 6.38 (s, 1H), 6.22 (s, 1H), 4.29 (q, J = 7.07 Hz, 2H), 3.62 (s, 4H), 3.08 (s, 4H), 2.75 (s, 2H), 2.20‐2.15 (m, 5H), 1.97 (s, 2H), 1.40 (t, J = 5.8 Hz, 2H), 1.33‐1.25 (m, 4H), 0.94 (s, 6H). This product was dissolved in a solution of potassium hydroxide in hydroxylamine (0.14 M) and stirred at room temperature for 1 h. The reaction was diluted in ethyl acetate and partitioned with saturated ammonium chloride. The product was extracted into ethyl acetate. The organic layers were combined, dried over sodium sulfate, filtered and concentrated to dryness. The product was isolated via flash column chromatography over silica gel eluting with a mixture of CH_2_Cl_2_/MeOH/H_2_O, 79:9:1, delivering 2‐((2‐((4‐(*N*‐(2‐((1*H*‐pyrrolo[2,3‐b]pyridin‐5‐yl)oxy)‐4‐(4‐((4′‐chloro‐5,5‐dimethyl‐3,4,5,6‐tetrahydro‐[1,1′‐biphenyl]‐2‐yl)methyl)piperazin‐1‐yl)benzoyl)sulfamoyl)‐2‐nitrophenyl)amino)ethyl)amino)‐N‐hydroxypyrimidine‐5‐carboxamide (**BD‐4‐208**) as a yellow solid (yield = 71%): ^1^H NMR(400 MHz, *d*_*6*_‐DMSO) δ11.63 (1H, s), 11.10 (1H, s), 9.02 (1H, s), 8.69 (3H, m), 8.49 (1H, s), 8.00 (2H, s), 7.76 (1H, d, *J* = 8.0 Hz), 7.55 (1H, d, *J* = 8.8 Hz), 7.41 (5H, m), 7.06 (2H, d, *J* = 7.6 Hz), 6.67 (1H, d, *J* = 8.8 Hz), 6.35 (1H, s), 6.23 (1H, s), 3.59 (4H, s), 3.06 (4H, s), 2.75 (2H, s), 2.20 (7H, m), 1.98 (2H, s), 1.41 (2H, app. t), 1.27 (4H, m), 0.95 (6H, s); HRMS (ESI) m/z [M + H]+ calcd for C_46_H_48_ClN_11_O_8_S = 950.3169; found = 950.3164.

Synthesis of (*E*)‐*2‐((1H‐pyrrolo[2,3‐b]pyridin‐5‐yl)oxy)‐4‐(4‐((4′‐chloro‐5,5‐dimethyl‐3,4,5,6‐tetrahydro‐[1,1′‐biphenyl]‐2‐yl)methyl)piperazin‐1‐yl)‐N‐((4‐((2‐(4‐(3‐(hydroxyamino)‐3‐oxoprop‐1‐en‐1‐yl)benzamido)ethyl)amino)‐3‐nitrophenyl)sulfonyl)benzamide (**SF‐8‐062**)*:

*Methyl (E)‐3‐(4‐((2‐((tert‐butoxycarbonyl)amino)ethyl)carbamoyl)phenyl)acrylate (**22**):* (*E*)‐4‐(3‐methoxy‐3‐oxoprop‐1‐en‐1‐yl)benzoic acid (2.91 mmol, 1.0 eq) was dissolved in DMF (0.1 M). Mono‐Boc‐ethylenediamine (3.49 mmol, 1.1 eq) was added, followed by HBTU (3.39 mmol, 1.1 eq) and DMAP (0.291 mmol, 0.1 eq). The reaction mixture was stirred at room temperature for 16 h. Upon completion, the reaction mixture was diluted in ethyl acetate and partitioned with water. The product was extracted into ethyl acetate (3x). The organic layers were combined and washed with 1 M HCl, saturated sodium bicarbonate, water (3x) and saturated sodium chloride, then dried over sodium sulfate, filtered and concentrated to dryness, yielding methyl (*E*)‐3‐(4‐((2‐((*tert*‐butoxycarbonyl)amino)ethyl)carbamoyl)phenyl)acrylate (**2**) as a tan solid that needed no further purification: (yield = 50%): ^1^H NMR (400 MHz, *d*_*6*_‐DMSO) δ 8.52 (1H, app. t), 7.84 (2H, d, *J* = 8.8 Hz), 7.79 (2H, d, *J* = 8.8 Hz), 7.67 (1H, d, *J* = 16.4 Hz), 6.92 (1H, t, *J* = 4.8 Hz), 6.73 (1H, d, *J* = 16.4 Hz), 3.72 (3H, s), 3.26 (2H, q, *J* = 6.4 Hz), 3.08 (q, *J* = 5.2 Hz), 1.35 (9H, s).

*Methyl (E)‐3‐(4‐((2‐((2‐nitro‐4‐sulfamoylphenyl)amino)ethyl)carbamoyl)phenyl)acrylate (**23**):* Methyl (*E*)‐3‐(4‐((2‐((*tert*‐butoxycarbonyl)amino)ethyl)carbamoyl)phenyl)acrylate (**22**; 1.55 mmol) was deprotected according to *General Procedure B*, resulting in methyl (*E*)‐3‐(4‐((2‐aminoethyl)carbamoyl)phenyl)acrylate trifluoroacetate without further purification. (tan solid, yield = 99%). Subsequently, methyl (*E*)‐3‐(4‐((2‐aminoethyl)carbamoyl)phenyl)acrylate trifluoroacetate (1.55 mmol) was reacted according to *General Procedure E*, yielding methyl (*E*)‐3‐(4‐((2‐((2‐nitro‐4‐sulfamoylphenyl)amino)ethyl)carbamoyl)phenyl)acrylate (**23**) as a yellow solid that required no further purification (yield = 99%): ^1^H NMR (400 MHz, *d*_*6*_‐DMSO) δ8.78 (1H, app. t), 8.69 (1H, t, *J* = 4.8 Hz), 8.45 (1H, s), 7.82 (5H, m), 7.65 (1H, d, *J* = 16.4 Hz), 7.35 (3H, m), 6.73 (1H, d, *J* = 16.4 Hz), 3.72 (3H, s), 3.61 (2H, q, 5.6 Hz), 3.50 (2H, q, 5.6 Hz).

*Methyl (E)‐3‐(4‐((2‐((4‐(N‐(2‐((1H‐pyrrolo[2,3‐b]pyridin‐5‐yl)oxy)‐4‐(4‐((4′‐chloro‐5,5‐dimethyl‐3,4,5,6‐tetrahydro‐[1,1′‐biphenyl]‐2‐yl)methyl)piperazin‐1‐yl)benzoyl)sulfamoyl)‐2‐nitrophenyl)amino)ethyl)carbamoyl)phenyl)acrylate (**24**):* Methyl (*E*)‐3‐(4‐((2‐((2‐nitro‐4‐sulfamoylphenyl)amino)ethyl)carbamoyl)phenyl)acrylate (**23**; 0.175 mmol) was reacted with **7** according to *General Procedure C*, resulting in methyl (*E*)‐3‐(4‐((2‐((4‐(N‐(2‐((1*H*‐pyrrolo[2,3‐b]pyridin‐5‐yl)oxy)‐4‐(4‐((4′‐chloro‐5,5‐dimethyl‐3,4,5,6‐tetrahydro‐[1,1′‐biphenyl]‐2‐yl)methyl)piperazin‐1‐yl)benzoyl)sulfamoyl)‐2‐nitrophenyl)amino)ethyl)carbamoyl)phenyl)acrylate (**24**) as a yellow solid that required no further purification (yield = 99%): ^1^H NMR (400 MHz, *d*_*6*_‐DMSO) δ 11.65 (1H, s), 8.76 (1H, app. t), 8.71 (1H, app. t), 8.53 (1H, s), 8.01 (1H, s), 7.81 (5H, m), 7.66 (1H, d, *J* = 16.0 Hz), 7.48 (3H, m), 7.32 (2H, d, *J* = 8.0 Hz), 7.17 (1H, d, *J* = 9.2 Hz), 7.02 (2H, d, *J* = 8.0 Hz), 6.72 (1H, d, *J* = 16.4 Hz), 6.64 (1H, d, *J* = 9.6 Hz), 6.36 (1H, s), 6.17 (1H, s), 3.72 (3H, s), 3.59 (2H, m), 3.51 (2H, m), 3.04 (4H, s), 2.72 (2H, s), 2.16 (6H, m), 1.93 (2H, s), 1.37 (2H, t, *J* = 5.2 Hz), 0.91 (6H. s); ^13^C NMR (500 MHz, *d*_*6*_‐DMSO) δ 167.08, 166.90, 158.34, 155.03, 147.84, 147.31, 145.97, 144.05, 142.60, 137.24, 134.17, 135.87, 134.94, 134.52, 132.75, 131.43, 130.61, 130.41, 129.39, 128.87, 128.67, 128.31, 128.10, 120.37, 120.08, 118.34, 115.33, 109.35, 103.20, 100.54, 60.28, 52.66, 52.18, 47.22, 26.91, 42.79, 38.83, 35.43, 29.47, 28.51, 25.76.

*(E)‐3‐(4‐((2‐((4‐(N‐(2‐((1H‐pyrrolo[2,3‐b]pyridin‐5‐yl)oxy)‐4‐(4‐((4′‐chloro‐5,5‐dimethyl‐3,4,5,6‐tetrahydro‐[1,1′‐biphenyl]‐2‐yl)methyl)piperazin‐1‐yl)benzoyl)sulfamoyl)‐2‐nitrophenyl)amino)ethyl)carbamoyl)phenyl)acrylic acid (**25**):* **24** (0.329 mmol) was saponified according to *General Procedure D*, delivering (*E*)‐3‐(4‐((2‐((4‐(N‐(2‐((1H‐pyrrolo[2,3‐b]pyridin‐5‐yl)oxy)‐4‐(4‐((4′‐chloro‐5,5‐dimethyl‐3,4,5,6‐tetrahydro‐[1,1′‐biphenyl]‐2‐yl)methyl)piperazin‐1‐yl)benzoyl)sulfamoyl)‐2‐nitrophenyl)amino)ethyl)carbamoyl)phenyl)acrylic acid (**25**) as a yellow solid (yield = 88%): ^1^H NMR: (400 MHz, *d*_*6*_‐DMSO) δ 11.68 (1H, s), 8.76 (2H, m), 8.56 (1H, s), 8.03 (1H, s), 7.82 (3H, m), 7.76 (2H, d, *J* = 8.0 Hz), 7.59 (1H, d, *J* = 16.4 Hz), 7.54 (1H, s), 7.47 (2H, d, *J* = 9.6 Hz), 7.33 (2H, d, *J* = 8.0 Hz), 7.21 (1H, d, *J* = 9.2 Hz), 7.03 (2H, d, *J* = 7.6 Hz), 6.66 (1H, d, *J* = 9.2 Hz), 6.60 (1H, d, *J* = 15.6 Hz), 6.37 (1H, s), 6.18 (1H, s), 6.60 (2H, m), 3.51 (2H, m), 3.11 (4H, m), 3.36 (2H, m), 3.31 (3H, m), 2.15 (2H, app. t), 1.95 (3H, m), 1.38 (2H, t, *J* = 5.2 Hz), 1.22 (1H, m), 0.91 (6H, s); ^13^C NMR (500 MHz, *d*_*6*_‐DMSO) δ 181.34, 167.99, 166.92, 158.51, 146.07, 143.43, 137.53, 135.91, 130.83, 128.72, 128.30, 121.53, 120.39, 119.99, 118.55, 109.89, 120.98, 100.59, 88.13, 49.76, 46.99, 35.34, 29.46, 28.53, 25.77.

*(E)‐2‐((1H‐pyrrolo[2,3‐b]pyridin‐5‐yl)oxy)‐4‐(4‐((4'‐chloro‐5,5‐dimethyl‐3,4,5,6‐tetrahydro‐[1,1'‐biphenyl]‐2‐yl)methyl)piperazin‐1‐yl)‐N‐((3‐nitro‐4‐((2‐(4‐(3‐oxo‐3‐(((tetrahydro‐2H‐pyran‐2‐yl)oxy)amino)prop‐1‐en‐1‐yl)benzamido)ethyl)amino)phenyl)sulfonyl)benzamide (**26**).* Compound **25** (0.253 mmol) was reacted with *O‐*THP‐hydroxylamine according to *General Procedure A*. Upon completion, the reaction mixture was diluted in ethyl acetate and partitioned with water. The product was extracted into ethyl acetate (3x). The organic layers were combined and washed with water (3x) and saturated sodium chloride, then dried over sodium sulfate, filtered and concentrated to dryness, yielding (*E*)‐2‐((1H‐pyrrolo[2,3‐b]pyridin‐5‐yl)oxy)‐4‐(4‐((4′‐chloro‐5,5‐dimethyl‐3,4,5,6‐tetrahydro‐[1,1′‐biphenyl]‐2‐yl)methyl)piperazin‐1‐yl)‐*N*‐((3‐nitro‐4‐((2‐(4‐(3‐oxo‐3‐(((tetrahydro‐2*H*‐pyran‐2‐yl)oxy)amino)prop‐1‐en‐1‐yl)benzamido)ethyl)amino)phenyl)sulfonyl)benzamide (**26**) as a yellow solid that was not purified further. Crude (*E*)‐2‐((1H‐pyrrolo[2,3‐b]pyridin‐5‐yl)oxy)‐4‐(4‐((4′‐chloro‐5,5‐dimethyl‐3,4,5,6‐tetrahydro‐[1,1′‐biphenyl]‐2‐yl)methyl)piperazin‐1‐yl)‐*N*‐((3‐nitro‐4‐((2‐(4‐(3‐oxo‐3‐(((tetrahydro‐2*H*‐pyran‐2‐yl)oxy)amino)prop‐1‐en‐1‐yl)benzamido)ethyl)amino)phenyl)sulfonyl)benzamide (**26**; 0.147 mmol) was reacted according to *General Procedure G*, resulting in (*E*)‐2‐((1*H*‐pyrrolo[2,3‐b]pyridin‐5‐yl)oxy)‐4‐(4‐((4′‐chloro‐5,5‐dimethyl‐3,4,5,6‐tetrahydro‐[1,1′‐biphenyl]‐2‐yl)methyl)piperazin‐1‐yl)‐N‐((4‐((2‐(4‐(3‐(hydroxyamino)‐3‐oxoprop‐1‐en‐1‐yl)benzamido)ethyl)amino)‐3‐nitrophenyl)sulfonyl)benzamide (**SF‐8‐062**) as a yellow solid after flash column chromatography over silica gel eluting with a mixture of CH_2_Cl_2_/MeOH/AcOH, 75:21:1 (yield = 63%): ^1^H NMR (400 MHz, *d*_*6*_‐DMSO) δ 11.68 (1H, s), 10.81 (1H, s), 9.08 (1H, s), 8.75 (1H, app. t), 8.56 (1H, s), 8.03 (1H, s), 7.83 (3H, m), 7.62 (2H, d, *J* = 7.6 Hz), 7.49 (5H, m), 7.34 (2H, d, *J* = 8.0 Hz), 7.22 (2H, d, *J* = 9.2 Hz), 7.04 (2H, d, *J* = 7.6 Hz), 6.67 (1H, d, *J* = 7.6 Hz), 6.53 (1H, d, *J* = 15.6 Hz), 6.38 (1H, s), 6.19 (1H, s), 3.59 (3H, m), 3.52 (4H, m), 3.07 (4H, m), 2.17 (3H, m), 1.95 (3H, m), 1.39 (5H, m), 1.22 (9H, m), 0.92 (8H, s), 0.84 (6H, m); ^13^C NMR (500 MHz, *d*_*6*_‐DMSO) δ 166.97, 164.32, 163.03, 161.02, 158.39, 147.96, 147.07, 146.00, 141.97, 138.17, 137.79, 135.81, 135.19, 134.41, 132.73, 132.12, 131.79, 130.51, 130.38, 128.91 (2), 128.43, 127.91 (2), 121.60, 120.38, 119.08, 118.40, 115.54, 109.50, 103.35, 100.54, 59.39, 51.93, 47.06, 42.91, 38.70, 35.23, 29.35, 28.49 (2), 25.99, 22.66, 14.46; HRMS (ESI) m/z [M + H]+ calcd for C_51_H_52_ClN_9_O_9_S = 1002.3370; found = 1002.3371.

Synthesis of *N* ^*1*^ *‐(2‐((4‐(N‐(2‐((1H‐pyrrolo[2,3‐b]pyridin‐5‐yl)oxy)‐4‐(4‐((4′‐chloro‐5,5‐dimethyl‐3,4,5,6‐tetrahydro‐[1,1′‐biphenyl]‐2‐yl)methyl)piperazin‐1‐yl)benzoyl)sulfamoyl)‐2‐nitrophenyl)amino)ethyl)‐N* ^*4*^ *‐hydroxyterephthalamide (**SF‐8‐083**):*

Mono‐Boc‐ethylenediamine (5.67 mmol) was reacted with **5** according to *General Procedure E*, resulting in *tert*‐butyl (2‐((2‐nitro‐4‐sulfamoylphenyl)amino)ethyl)carbamate that was used without further purification. Crude *tert*‐butyl (2‐((2‐nitro‐4‐sulfamoylphenyl)amino)ethyl)carbamate (1.0 mmol) was reacted according to *General Procedure C*, resulting in *tert*‐butyl (2‐((4‐(*N*‐(2‐((1*H*‐pyrrolo[2,3‐b]pyridin‐5‐yl)oxy)‐4‐(4‐((4′‐chloro‐5,5‐dimethyl‐3,4,5,6‐tetrahydro‐[1,1′‐biphenyl]‐2‐yl)methyl)piperazin‐1‐yl)benzoyl)sulfamoyl)‐2‐nitrophenyl)amino)ethyl)carbamate that needed no further purification (**27**): ^1^H NMR (400 MHz, DMSO) δ = 11.65 (s, 1H), 8.60 (s, 1H), 8.53 (s, 1H), 8.02 (s, 1H), 7.78 (d, J = 9.2 Hz, 1H), 7.51–7.48 (m, 3H), 7.33 (d, J = 8.0 Hz, 2H), 7.09–7.01 (m, 4H), 6.66 (d, J = 8.8 Hz, 1H), 6.37 (s, 1H), 6.18 (s, 1H), 3.43 (s, 2H), 3.18 (s, 2H), 3.05 (s, 4H), 2.72 (s, 2H), 2.17–2.13 (m, 6H), 1.93 (s, 2H), 1.37–1.27 (m, 11H), 0.91 (s, 6H).

Subsequently, tert‐butyl (2‐((4‐(N‐(2‐((1H‐pyrrolo[2,3‐b]pyridin‐5‐yl)oxy)‐4‐(4‐((4′‐chloro‐5,5‐dimethyl‐3,4,5,6‐tetrahydro‐[1,1′‐biphenyl]‐2‐yl)methyl)piperazin‐1‐yl)benzoyl)sulfamoyl)‐2‐nitrophenyl)amino)ethyl)carbamate (**27**; 0.515 mmol) was dissolved in CH_2_Cl_2_ (5 mL). 4 M HCl in dioxane (5 mL) was added and the reaction mixture was stirred at room temperature for 4 h. Upon completion the reaction mixture was concentrated to dryness and azeotroped with CHCl_3_ and methanol (3x), resulting in 2‐((1H‐pyrrolo[2,3‐b]pyridin‐5‐yl)oxy)‐N‐((4‐((2‐aminoethyl)amino)‐3‐nitrophenyl)sulfonyl)‐4‐(4‐((4′‐chloro‐5,5‐dimethyl‐3,4,5,6‐tetrahydro‐[1,1′‐biphenyl]‐2‐yl)methyl)piperazin‐1‐yl)benzamide hydrochloride (**28**) that was advanced without further purification.

Separately, mono‐methyl terephthalate (18.8 mmol) was reacted with *O‐*THP‐hydroxylamine (17.08 mmol) according to *General Procedure A*. Upon completion, the reaction mixture was diluted in ethyl acetate and partitioned with water. The product was extracted into ethyl acetate (3x). The organic layers were combined and washed with 1 M HCl, saturated sodium bicarbonate, water (3x) and saturated sodium chloride, then dried over sodium sulfate and concentrated to dryness. The product was isolated via flash column chromatography over silica gel eluting with a mixture of 50% hexanes in ethyl acetate, yielding methyl 4‐(((tetrahydro‐2*H*‐pyran‐2‐yl)oxy)carbamoyl)benzoate as a pale yellow oil (yield = 98%), whose spectral data were consistent with published data (Schneider et al. 2016). Methyl 4‐(((tetrahydro‐2*H*‐pyran‐2‐yl)oxy)carbamoyl)benzoate (20.30 mmol) was reacted according to *General Procedure D*, resulting in 4‐(((tetrahydro‐2H‐pyran‐2‐yl)oxy)carbamoyl)benzoic acid (**29**) as a white solid (yield = 62%), whose spectral data were consistent with published data (Schneider et al. 2016). 2‐((1H‐pyrrolo[2,3‐b]pyridin‐5‐yl)oxy)‐N‐((4‐((2‐aminoethyl)amino)‐3‐nitrophenyl)sulfonyl)‐4‐(4‐((4′‐chloro‐5,5‐dimethyl‐3,4,5,6‐tetrahydro‐[1,1′‐biphenyl]‐2‐yl)methyl)piperazin‐1‐yl)benzamide hydrochloride (**28**; 0.163 mmol) was reacted with 4‐(((tetrahydro‐2H‐pyran‐2‐yl)oxy)carbamoyl)benzoic acid (**29**; 0.163 mmol) according to *General Procedure A*. Upon completion, the reaction mixture was diluted in ethyl acetate and partitioned with water. The product was extracted into ethyl acetate (3x). The organic layers were combined and washed with water (3x) and saturated sodium chloride, then dried over sodium sulfate and concentrated to dryness. The product was isolated via flash column chromatography over silica gel eluting with a mixture of CH_2_Cl_2_/MeOH/NH_4_OH, 92:7:1, yielding *N* ^1^‐(2‐((4‐(*N*‐(2‐((1*H*‐pyrrolo[2,3‐b]pyridin‐5‐yl)oxy)‐4‐(4‐((4′‐chloro‐5,5‐dimethyl‐3,4,5,6‐tetrahydro‐[1,1′‐biphenyl]‐2‐yl)methyl)piperazin‐1‐yl)benzoyl)sulfamoyl)‐2‐nitrophenyl)amino)ethyl)‐*N* ^4^‐((tetrahydro‐2H‐pyran‐2‐yl)oxy)terephthalamide (**30**) as a yellow solid (yield = 75%): ^1^H NMR (400 MHz, *d*_6_‐DMSO) δ 11.75 (s, 1H), 11.68 (s, 1H), 8.84 (s, 1H), 8.76 (s, 1H), 8.56 (s, 1H), 8.03 (s, 1H), 7.89 (d, J = 8.8 Hz, 2H), 7.83 (d, J = 7.6 hz, 3H), 7.54 (s, 1H), 7.48 (d, J = 9.6 Hz, 2H), 7.35 (d, J = 8.0 Hz, 2H), 7.23 (d, J = 9.2 Hz, 1H), 7.05 (d, J = 8.4 Hz, 2H), 6.68 (d, J = 8.4 Hz, 1H), 6.38 (s, 1H), 6.21 (s, 1H), 4.13 (s, 1H), 3.60 (s, 2H), 3.53 (s, 4H), 3.31 (s, 6H), 2.49 (s, 4H), 2.19 (s, 2H), 2.00 (s, 2H), 1.71 (s, 2H), 1.54 (s, 2H), 1.40 (s, 2H), 1.22 (s, 2H), 0.92 (s, 6H). *N* ^1^‐(2‐((4‐(*N*‐(2‐((1*H*‐pyrrolo[2,3‐b]pyridin‐5‐yl)oxy)‐4‐(4‐((4′‐chloro‐5,5‐dimethyl‐3,4,5,6‐tetrahydro‐[1,1′‐biphenyl]‐2‐yl)methyl)piperazin‐1‐yl)benzoyl)sulfamoyl)‐2‐nitrophenyl)amino)ethyl)‐*N* ^4^‐((tetrahydro‐2H‐pyran‐2‐yl)oxy)terephthalamide (**30**; 0.09 mmol) was reacted according to *General Procedure G*. The product was extracted into ethyl acetate (3x). The organic layers were combined and washed with water (3x) and saturated sodium chloride, then dried over sodium sulfate and concentrated to dryness. The product was isolated via flash column chromatography over silica gel eluting with a mixture of CH_2_Cl_2_/MeOH/H_2_O, 79:9:1, yielding *N* ^1^‐(2‐((4‐(N‐(2‐((1H‐pyrrolo[2,3‐b]pyridin‐5‐yl)oxy)‐4‐(4‐((4′‐chloro‐5,5‐dimethyl‐3,4,5,6‐tetrahydro‐[1,1′‐biphenyl]‐2‐yl)methyl)piperazin‐1‐yl)benzoyl)sulfamoyl)‐2‐nitrophenyl)amino)ethyl)‐*N* ^4^‐hydroxyterephthalamide (**SF‐8‐083**) as a yellow solid (yield = 91%): ^1^H NMR (400 MHz, *d*_*6*_‐DMSO) δ 11.68 (1H, s), 11.34 (1H, s), 9.12 (1H, s), 8.86 (1H, app. t), 8.72 (1H, app, t), 8.54 (1H, s), 8.02 (1H, s), 7.87 (2H, d, *J* = 8.0 Hz), 7.81 (3H, m), 7.49 (2H, m), 7.32 (2H, d, *J* = 8.0 Hz), 7.20 (1H, d, *J* = 9.6 Hz), 7.03 (2H, d, *J* = 7.6 Hz), 6.67 (1H, d, *J* = 8.4 Hz), 6.36 (1H, s), 6.18 (1H, s), 3.59 (2H, m), 3.53 (2H, m), 3.09 (4H, m), 2.77 (1H, m), 2.23 (6H, m), 1.94 (2H, s), 1.37 (2H, app. t), 1.22 (1H, s), 0.91 (6H, s); HRMS (ESI) m/z [M + H]+ calcd for C_49_H_50_ClN_9_O_9_S = 976.3214; found = 976.3219.

Synthesis of *2‐((1H‐pyrrolo[2,3‐b]pyridin‐5‐yl)oxy)‐N‐((4‐((4‐((2‐amino‐4‐(thiophen‐2‐yl)phenyl)carbamoyl)benzyl)amino)‐3‐nitrophenyl)sulfonyl)‐4‐(4‐((4′‐chloro‐5,5‐dimethyl‐3,4,5,6‐tetrahydro‐[1,1′‐biphenyl]‐2‐yl)methyl)piperazin‐1‐yl)benzamide (**AMC‐4‐074**):* To a solution of 4‐bromo‐nitroaniline (9.2 mmol, 1.0 eq) in THF (0.2 M), Boc_2_O (23 mmol, 2.5 eq) and DMAP (0.92 mmol, 0.1 eq) were added and the reaction was stirred at room temperature for 16 h. Upon completion, the reaction mixture was concentrated to dryness and dissolved in CH_2_Cl_2_ (0.1 M), and TFA (27.6 mmol, 3.0 eq) was added to effect mono‐Boc‐deprotection. The reaction was left to stir at room temperature for 72 h. Upon completion, the reaction mixture was partitioned between CH_2_Cl_2_ and water. The organic layer was washed with water (3x) and saturated sodium chloride, then dried over sodium sulfate and concentrated to dryness. The product was isolated flash chromatography on silica gel using a gradient of ethyl acetate in hexanes, yielding *tert‐*butyl (4‐bromo‐2‐nitrophenyl)carbamate (yellow solid, yield = 99%) (Reis et al. 2016). To a solution of *tert‐*butyl (4‐bromo‐2‐nitrophenyl)carbamate (1.58 mmol, 1.0 eq) in dioxane/H_2_O, 4:1 (0.05 M), thiothene‐2yl‐boronic acid (3.08 mmol, 1.95 eq) and potassium phosphate (4.66 mmol, 2.95 eq) were added. The flask was evacuated then flushed with nitrogen. Tetrakis(triphenylphosphine)palladium (0.159 mmol, 0.1 eq) was added and nitrogen was bubbled through the solution for 15 min. The reaction was refluxed at 110°C for 16 h. Upon completion, the reaction mixture was cooled to room temperature and filtered through celite washing with ethyl acetate and concentrated to dryness. The product was isolated via flash chromatography over silica gel using a gradient of 9 hexane:1 ethyl acetate, yielding *tert*‐butyl (2‐nitro‐4‐(thiophen‐2‐yl)phenyl)carbamate as an orange solid (yield = 81%) (Reis et al. 2016). Next, *tert*‐butyl (2‐nitro‐4‐(thiophen‐2‐yl)phenyl)carbamate (1.125 mmol) was reacted according to *General Procedure F*, resulting in *tert*‐butyl (2‐amino‐4‐(thiophen‐2‐yl)phenyl)carbamate (orange solid) that was not purified further (yield = 82%). Crude *tert*‐butyl (2‐amino‐4‐(thiophen‐2‐yl)phenyl)carbamate (0.183 mmol, 1.1 eq) was reacted with acid **8** according to *General Procedure A*. The product was isolated via flash column chromatography over silica gel, eluting with a mixture of CH_2_Cl_2_/MeOH/NH_4_OH, 92:7:1, resulting in *tert*‐butyl (2‐(4‐(((4‐(N‐(2‐((1*H*‐pyrrolo[2,3‐b]pyridin‐5‐yl)oxy)‐4‐(4‐((4′‐chloro‐5,5‐dimethyl‐3,4,5,6‐tetrahydro‐[1,1′‐biphenyl]‐2‐yl)methyl)piperazin‐1‐yl)benzoyl)sulfamoyl)‐2‐nitrophenyl)amino)methyl)benzamido)‐4‐(thiophen‐2‐yl)phenyl)carbamate (**31**) as a yellow solid (yield = 77%). Subsequently, *tert*‐butyl (2‐(4‐(((4‐(N‐(2‐((1*H*‐pyrrolo[2,3‐b]pyridin‐5‐yl)oxy)‐4‐(4‐((4′‐chloro‐5,5‐dimethyl‐3,4,5,6‐tetrahydro‐[1,1′‐biphenyl]‐2‐yl)methyl)piperazin‐1‐yl)benzoyl)sulfamoyl)‐2‐nitrophenyl)amino)methyl)benzamido)‐4‐(thiophen‐2‐yl)phenyl)carbamate (**31**; 0.059 mmol) was dissolved in CH_2_Cl_2_ (1 mL). TFA (1 mL) was added, and the reaction was stirred at room temperature for 30 min. Upon completion, the reaction mixture was diluted with CH_2_Cl_2_ and partitioned with water. The aqueous layer was basified with saturated sodium bicarbonate and the product was extracted into CH_2_Cl_2_ (2x). The organic layers were combined and dried over sodium sulfate, filtered and concentrated to dryness, delivering 2‐((1*H*‐pyrrolo[2,3‐b]pyridin‐5‐yl)oxy)‐*N*‐((4‐((4‐((2‐amino‐4‐(thiophen‐2‐yl)phenyl)carbamoyl)benzyl)amino)‐3‐nitrophenyl)sulfonyl)‐4‐(4‐((4′‐chloro‐5,5‐dimethyl‐3,4,5,6‐tetrahydro‐[1,1′‐biphenyl]‐2‐yl)methyl)piperazin‐1‐yl)benzamide (**AMC‐4‐074**) as a yellow solid without further purification (yield = 99%): ^1^H NMR (400 MHz, *d*_6_‐DMSO) δ 11.71(1H, s), 9.67 (1H, s), 9.20 (1H, s), 8.60 (1H, s), 8.02 (1H, s), 7.96 (2H, d, *J* = 7.6 Hz), 7.75 (1H, d, *J* = 8.4 Hz), 7.52 (6H, m), 7.34 (3H, m), 7.28 (1H, d, *J* = 7.6 Hz,), 7.21 (1H, s), 7.04 (3H, d, *J* = 7.6 Hz), 6.89 (1H, d, *J* = 9.6 Hz), 6.79 (1H, d, *J* = 8 Hz), 6.17 (1H, d), 6.39 (1H, s), 6.19 (1H, s), 5.15 (2H, s), 4.75 (2H, s), 3.31 (2H, s), 2.16 (2H, s), 1.97 (2H, s), 0.91 (6H, s); ^13^C NMR (500 MHz, *d*_*6*_‐DMSO) δ 165.87, 164.11, 158.51, 154.66, 147.65, 146.93, 146.14, 144.86, 143.60, 141.93, 135.90, 134.42, 134.16, 132.78, 131.92, 130.82, 130.47 (2), 129.00, 128.87, 128.81, 128.45 (2), 127.38 (2), 125.64, 124.53 (2), 123.99, 123.80, 122.85, 121.60, 120.45, 118.64, 116.96, 155.86, 109.55, 103.15, 100.63, 59.28, 51.81, 47.07, 46.27, 45.55, 35.13, 29.35 (2), 28.49 (2), 25.70.

Synthesis of *2‐((1H‐pyrrolo[2,3‐b]pyridin‐5‐yl)oxy)‐N‐((4‐((4‐((3‐amino‐[1,1′‐biphenyl]‐4‐yl)carbamoyl)benzyl)amino)‐3‐nitrophenyl)sulfonyl)‐4‐(4‐((4′‐chloro‐5,5‐dimethyl‐3,4,5,6‐tetrahydro‐[1,1′‐biphenyl]‐2‐yl)methyl)piperazin‐1‐yl)benzamide (**AMC‐4‐075**):* To a solution of 4‐bromo‐nitroaniline (9.2 mmol, 1.0 eq) in THF (0.2 M), Boc_2_O (23 mmol, 2.5 eq) and DMAP (0.92 mmol, 0.1 eq) were added and the reaction was stirred at room temperature for 16 h. Upon completion, the reaction mixture was concentrated to dryness and dissolved in CH_2_Cl_2_ (0.1 M), TFA (27.6 mmol, 3.0 eq) was added to effect mono‐Boc‐deprotection. The reaction was left to stir at room temperature for 72 h. Upon completion, the reaction mixture was partitioned between CH_2_Cl_2_ and water. The organic layer was washed with water (x3) and saturated sodium chloride, then dried over sodium sulfate and concentrated to dryness. The product was isolated via flash column chromatography over silica gel using a gradient of ethyl acetate in hexanes, yielding *tert‐*butyl (4‐bromo‐2‐nitrophenyl)carbamate as a yellow solid (yield = 99%) (Itoh et al. 2023). To a solution of *tert‐*butyl (4‐bromo‐2‐nitrophenyl)]carbamate (1.58 mmol, 1.0 eq) in dioxane/H_2_O, 4:1, phenyl boronic acid (3.08 mmol, 1.95 eq) and potassium phosphate (4.66 mmol, 2.95 eq) were added. The flask was evacuated then flushed through with nitrogen. Tetrakis(triphenylphosphine)palladium (0.159 mmol, 0.1 eq) was added and nitrogen was bubbled through the solvent for 10 min, after which the reaction was refluxed at 110°C for 16 h. The reaction mixture was cooled to room temperature then filtered through celite, rinsing with ethyl acetate, and concentrated to dryness. The product was isolated via flash chromatography on a silica gel using a gradient of 9 hexane:1 ethyl acetate, resulting in *tert*‐butyl (3‐nitro‐[1,1′‐biphenyl]‐4‐yl)carbamate as an orange solid (yield = 70%) (Itoh et al. 2023).

*Tert*‐butyl (3‐nitro‐[1,1′‐biphenyl]‐4‐yl)carbamate (0.940 mmol) was reacted according to *General Procedure F*. The product was isolated via flash column chromatography over silica gel using a gradient of ethyl acetate in hexanes, resulting in *tert*‐butyl (3‐amino‐[1,1′‐biphenyl]‐4‐yl)carbamate as an orange solid (yield = 32%). *Tert*‐butyl (3‐amino‐[1,1′‐biphenyl]‐4‐yl)carbamate (0.183 mmol) was reacted with acid **8** according to *General Procedure A*. The product was isolated via flash column chromatography over silica gel, eluting with a mixture of CH_2_Cl_2_/MeOH/NH_4_OH, 92:7:1, resulting in *tert*‐butyl (3‐(4‐(((4‐(*N*‐(2‐((1*H*‐pyrrolo[2,3‐b]pyridin‐5‐yl)oxy)‐4‐(4‐((4′‐chloro‐5,5‐dimethyl‐3,4,5,6‐tetrahydro‐[1,1′‐biphenyl]‐2‐yl)methyl)piperazin‐1‐yl)benzoyl)sulfamoyl)‐2‐nitrophenyl)amino)methyl)benzamido)‐[1,1′‐biphenyl]‐4‐yl)carbamate (**32**) as a yellow solid (yield = 62%). *Tert*‐butyl (3‐(4‐(((4‐(*N*‐(2‐((1*H*‐pyrrolo[2,3‐b]pyridin‐5‐yl)oxy)‐4‐(4‐((4′‐chloro‐5,5‐dimethyl‐3,4,5,6‐tetrahydro‐[1,1′‐biphenyl]‐2‐yl)methyl)piperazin‐1‐yl)benzoyl)sulfamoyl)‐2‐nitrophenyl)amino)methyl)benzamido)‐[1,1′‐biphenyl]‐4‐yl)carbamate (**32**; 0.051 mmol) was dissolved in CH_2_Cl_2_ (1 mL). TFA (1 mL) was added, and the reaction was stirred at room temperature for 30 min. Upon completion, the reaction mixture was diluted with CH_2_Cl_2_ and partitioned with water. The aqueous layer was basified with saturated sodium bicarbonate and the product extracted into CH_2_Cl_2_ (2x). The organic layers were combined and dried over sodium sulfate and concentrated to dryness. The product was isolated via preparatory thin layer chromatography over silica gel, eluting with a mixture of CH_2_Cl_2_/MeOH/AcOH, 75:21:1, affording 2‐((1*H*‐pyrrolo[2,3‐b]pyridin‐5‐yl)oxy)‐*N*‐((4‐((4‐((3‐amino‐[1,1′‐biphenyl]‐4‐yl)carbamoyl)benzyl)amino)‐3‐nitrophenyl)sulfonyl)‐4‐(4‐((4′‐chloro‐5,5‐dimethyl‐3,4,5,6‐tetrahydro‐[1,1′‐biphenyl]‐2‐yl)methyl)piperazin‐1‐yl)benzamide (**AMC‐4‐075**) as a yellow solid (yield = 58%): ^1^H NMR (400 MHz, *d*_6_‐DMSO) δ 11.65 (1H, s), 9.67 (1H, s), 9.23 (1H, s), 8.55 (1H, s), 7.99 (1H, s), 7.95 (2H, d, *J* = 7.6 Hz), 7.68 (1H, s), 7.51 (8H, m), 7.34 (5H, m), 7.21 (1H, t, *J* = 7.4 Hz), 7.02 (2H, d, *J* = 8 Hz), 6.84 (2H, d, *J* = 8 Hz), 6.64 (1H, d, *J* = 8.4 Hz), 6.36 (1H, s), 6.17 (1H, s), 5.07 (2H, s), 4.73 (2H, s), 3.31 (2H, s), 3.02 (4H, s), 2.71 (2H, s), 2.14 (6H, s), 1.93 (2H, s), 0.91 (6H, s); ^13^C NMR (500 MHz, *d*_*6*_‐DMSO) δ 169.73, 165.88, 158.18, 154.82, 147.22, 145.91, 143.37, 142.62, 142.02, 140.81, 136.90, 135.83, 135.25, 137.90, 134.57, 134.28, 132.75, 131.43, 130.73, 130.63, 129.38, 128.82, 128.68, 128.22, 127.85, 127.38, 126.59, 126.09, 125.35, 124.17, 120.37, 119.16, 118.19, 117.06, 115.55, 109.43, 103.43, 100.54, 70.68, 60.29, 52.69, 50.83, 47.31, 46.93, 46.24, 44.84, 43.02, 39.21, 35.44, 29.49, 28.53, 25.77.

Synthesis *of 4‐(4‐(((4‐(N‐(2‐((1H‐pyrrolo[2,3‐b]pyridin‐5‐yl)oxy)‐4‐(4‐((4′‐chloro‐5,5‐dimethyl‐3,4,5,6‐tetrahydro‐[1,1′‐biphenyl]‐2‐yl)methyl)piperazin‐1‐yl)benzoyl)sulfamoyl)‐2‐nitrophenyl)amino)methyl)piperidin‐1‐yl)‐3,5‐difluoro‐N‐hydroxybenzamide (**AMC‐4‐101**):* To a stirring solution of methyl 3,4,5‐trifluorobenzene carboxylate (2.63 mmol, 1.0 eq) in DMSO (0.1 M), 4‐(Boc‐aminomethyl)piperidine (2.63 mmol, 1.0 eq) and potassium carbonate (5.26 mmol, 2.0 eq) were added and the reaction mixture was stirred at 90°C for 16 h. Upon completion, the reaction mixture was cooled to room temperature, dissolved in ethyl acetate, and partitioned with water. The product was extracted into additional ethyl acetate (3x). The organic layers were combined and washed with 1 M HCl, water (3x), and saturated sodium chloride, then dried over sodium sulfate, filtered and concentrated to dryness, resulting in methyl 4‐(4‐(((*tert*‐butoxycarbonyl)amino)methyl)piperidin‐1‐yl)‐3,5‐difluorobenzoate (**33**) as a brown solid (yield = 84%): ^1^H NMR: (400 MHz, CDCl_3_) δ7.71 (1H, t, *J* = 7.0 Hz), 7.50 (2H, d, *J* = 8.8 Hz), 3.90 (3H, s), 3.44 (2H, d, *J* = 11.6 Hz), 3.15‐3.09 (4H, m), 1.76 (2H, d, *J* = 12.4 Hz), 1.51‐1.47 (10H, m), 1.41–1.35 (2H, m).) without further purification Methyl 4‐(4‐(((*tert*‐butoxycarbonyl)amino)methyl)piperidin‐1‐yl)‐3,5‐difluorobenzoate (**33**; 2.00 mmol) was reacted according to *General Procedure B*, resulting in methyl 4‐(4‐(aminomethyl)piperidin‐1‐yl)‐3,5‐difluorobenzoate trifluoroacetate (**34**) without further purification. (white solid, yield = 99%).

Methyl 4‐(4‐(aminomethyl)piperidin‐1‐yl)‐3,5‐difluorobenzoate trifluoroacetate (**34**; 0.700 mmol) was reacted according to *General Procedure E*. The product was isolated via flash column chromatography over silica gel eluting with a mixture of CH_2_Cl_2_/MeOH/NH_4_OH, 92:7:1, resulting in methyl 3,5‐difluoro‐4‐(4‐(((2‐nitro‐4‐sulfamoylphenyl)amino)methyl)piperidin‐1‐yl)benzoate (**35**; yellow solid, yield = 90% yield). Methyl 3,5‐difluoro‐4‐(4‐(((2‐nitro‐4‐sulfamoylphenyl)amino)methyl)piperidin‐1‐yl)benzoate (**35**; 0.350 mmol) was reacted according to *General Procedure C*. The product was isolated via flash column chromatography over silica gel eluting with CH_2_Cl_2_/MeOH/NH_4_OH, 25:7:1, resulting in methyl 4‐(4‐(((4‐(*N*‐(2‐((1*H*‐pyrrolo[2,3‐b]pyridin‐5‐yl)oxy)‐4‐(4‐((4′‐chloro‐5,5‐dimethyl‐3,4,5,6‐tetrahydro‐[1,1′‐biphenyl]‐2‐yl)methyl)piperazin‐1‐yl)benzoyl)sulfamoyl)‐2‐nitrophenyl)amino)methyl)piperidin‐1‐yl)‐3,5‐difluorobenzoate (**36**) as a yellow solid (yield = 93%): (400 MHz, *d*_*6*_‐DMSO) δ 8.61 (1H, s), 8.54 (1H, s), 8.02 (1H, s), 7.78 (1H, d, *J* = 9.2 Hz), 7.49 (5H, d, *J* = 9.2 Hz), 7.33 (2H, d, *J* = 8.4 Hz), 7.23 (1H, m), 7.02 (2H, d, *J* = 8.0 Hz), 6.66 (1H, d, *J* = 8.8 Hz), 6.37 (1H, s), 6.18 (1H, s), 3.81 (2H, s), 3.83–3.31 (5H, m), 3.09–3.05 (6H, m), 2.72 (2H, s), 2.49 (4H, s), 2.16 (6H, s), 1.93 (2H, s), 1.77 (2H, d, *J* = 13.6 Hz), 1.37 (4H, s), 1.16 (1H, m), 0.91 (6H, s).

Methyl 4‐(4‐(((4‐(*N*‐(2‐((1*H*‐pyrrolo[2,3‐b]pyridin‐5‐yl)oxy)‐4‐(4‐((4′‐chloro‐5,5‐dimethyl‐3,4,5,6‐tetrahydro‐[1,1′‐biphenyl]‐2‐yl)methyl)piperazin‐1‐yl)benzoyl)sulfamoyl)‐2‐nitrophenyl)amino)methyl)piperidin‐1‐yl)‐3,5‐difluorobenzoate (**36**; 0.289 mmol) was reacted according to *General Procedure D*, resulting in 4‐(4‐(((4‐(*N*‐(2‐((1*H*‐pyrrolo[2,3‐b]pyridin‐5‐yl)oxy)‐4‐(4‐((4′‐chloro‐5,5‐dimethyl‐3,4,5,6‐tetrahydro‐[1,1′‐biphenyl]‐2‐yl)methyl)piperazin‐1‐yl)benzoyl)sulfamoyl)‐2‐nitrophenyl)amino)methyl)piperidin‐1‐yl)‐3,5‐difluorobenzoic acid without further purification as a yellow solid (**37**; yield = 99%). 4‐(4‐(((4‐(*N*‐(2‐((1*H*‐pyrrolo[2,3‐b]pyridin‐5‐yl)oxy)‐4‐(4‐((4′‐chloro‐5,5‐dimethyl‐3,4,5,6‐tetrahydro‐[1,1′‐biphenyl]‐2‐yl)methyl)piperazin‐1‐yl)benzoyl)sulfamoyl)‐2‐nitrophenyl)amino)methyl)piperidin‐1‐yl)‐3,5‐difluorobenzoic acid (**37**; 0.147 mmol) was reacted with *O*‐THP‐hydroxylamine (0.161 mmol) according to *General Procedure A*. Upon completion, the reaction mixture was dissolved in ethyl acetate and partitioned with water. The product was extracted from the aqueous with additional ethyl acetate (3x). The organic layers were combined and washed with water (3x) and saturated sodium chloride, then dried over sodium sulfate, filtered and concentrated to dryness. The product was isolated via flash column chromatography on silica gel using a gradient of CH_2_Cl_2_/MeOH/NH_4_OH, 21:7:1, resulting in 4‐(4‐(((4‐(*N*‐(2‐((1*H*‐pyrrolo[2,3‐*b*]pyridin‐5‐yl)oxy)‐4‐(4‐((4′‐chloro‐5,5‐dimethyl‐3,4,5,6‐tetrahydro‐[1,1′‐biphenyl]‐2‐yl)methyl)piperazin‐1‐yl)benzoyl)sulfamoyl)‐2‐nitrophenyl)amino)methyl)piperidin‐1‐yl)‐3,5‐difluoro‐*N*‐((tetrahydro‐2*H*‐pyran‐2‐yl)oxy)benzamide (**38**) as a yellow solid (yield = 44%).

Next, 4‐(4‐(((4‐(*N*‐(2‐((1*H*‐pyrrolo[2,3‐*b*]pyridin‐5‐yl)oxy)‐4‐(4‐((4′‐chloro‐5,5‐dimethyl‐3,4,5,6‐tetrahydro‐[1,1′‐biphenyl]‐2‐yl)methyl)piperazin‐1‐yl)benzoyl)sulfamoyl)‐2‐nitrophenyl)amino)methyl)piperidin‐1‐yl)‐3,5‐difluoro‐*N*‐((tetrahydro‐2*H*‐pyran‐2‐yl)oxy)benzamide (**38**) was reacted according to *General Procedure G*. The crude product was dissolved in ethyl acetate and partitioned with water. The aqueous layer was basified with saturated sodium bicarbonate and extracted into additional ethyl acetate (2x). The organic layers were combined and dried over sodium sulfate then concentrated to dryness. The product was isolated via preparatory thin layer chromatography over silica gel, eluting with a mixture of 79 CH_2_Cl_2_:9 methanol: 1 water, resulting in 4‐(4‐(((4‐(*N*‐(2‐((1*H*‐pyrrolo[2,3‐b]pyridin‐5‐yl)oxy)‐4‐(4‐((4′‐chloro‐5,5‐dimethyl‐3,4,5,6‐tetrahydro‐[1,1′‐biphenyl]‐2‐yl)methyl)piperazin‐1‐yl)benzoyl)sulfamoyl)‐2‐nitrophenyl)amino)methyl)piperidin‐1‐yl)‐3,5‐difluoro‐*N*‐hydroxybenzamide (**AMC‐4‐101**) as a yellow solid (yield = 89%): ^1^H NMR (400 MHz, *d*_*6*_‐DMSO) δ 11.54 (1H, s), 11.21 (1H, s), 9.10 (1H, s), 8.42 (2H, s), 7.94 (1H, s), 7.61 (1H, s), 7.53 (1H, d, *J* = 7.6 Hz), 7.40 (3H, m), 7.33 (2H, d, *J* = 8.8 Hz), 6.12 (1H, d, *J* = 7.6 Hz), 6.31 (1H, s), 6.22 (1H, s), 3.02 (6H, m), 2.71 (2H, s), 2.17 (6H, m), 1.94 (2H, m), 1.76 (3H, m), 1.37 (4H, m), 1.22 (1H, m), 0.91 (6H, s); ^13^C NMR (500 MHz, *d*_*6*_‐DMSO) δ 165.74, 162.27, 158.11, 157.69, 156.17, 154.28, 147.23, 145.63, 142.65, 135.66, 134.89, 134.77, 132.72, 131.41, 130.63 (2), 130.00, 129.58, 128.67 (2), 127.94, 127.18, 120.29, 117.52, 114.96, 111.73, 111.55, 109.60, 100.41, 74.81, 60.33, 52.79, 51.26, 48.36, 47.59, 46.92, 35.45, 35.10, 30.73, 29.49, 28.52 (2), 25.79.

### 2.2 HDAC and BCL‐2 Assays

HDAC1 and HDAC6 IC_50_ values were determined by Nanosyn using fluorogenic assays (BPS Bioscience, catalogue numbers 50061 and 50076, respectively) following manufacturer's protocols. BCL‐2 IC_50_ values were determined by Nanosyn using a TR‐FRET assay (BPS Bioscience, catalogue number 50222) following manufacturer's instructions.

#### 2.2.1 AML Cell Lines

As described previously, MOLM14 and MV4;11 cell lines (both human AML cell lines harboring both mixed‐lineage‐leukemia (KMT2A) rearrangement and FLT3‐ITD mutations) were obtained from DSMZ (Branschweig, DEU) and ATCC (Manassas, VA), respectively (Goodis et al. 2024; Moses et al. 2021); MV4;11 BCL‐2 over‐expressing (OE) cells were generated via lentiviral transduction as described previously (Empty Vector AddGene #227688; BLC‐2 OE AddGene #227690) (Goodis et al. 2024).

Cell lines were cultured in RPMI 1640 media (Cellgro, # MDT‐10‐040‐CV) containing 10% fetal bovine serum (Gemini BioProducts #100‐106) and passaged twice per week to maintain cultures in log‐phase‐growth. Upon receipt or derivation, cell lines were expanded and multiple aliquots cryopreserved in DMSO‐containing medium in the vapor phase of liquid nitrogen. Cells were genetically authenticated and confirmed to be mycoplasma‐negative before cryopreservation. Cells from a given cryopreserved aliquot were cultured for no more than 3–4 months.

#### 2.2.2 Viable Cell Counts

Viable cells counts were obtained using a Nexcelcom K2 cell counter (#CS2‐0106) after manufacturer's recommended staining with acridine orange and propidium iodide.

#### 2.2.3 Cell‐Based Assays

Cell metabolism/viability was assessed by alamarBlue assays (Thermo Fisher, cat# DAL1100) and cell apoptosis/death by Annexin V/7‐aminoactinomycin D (AnnV/7AAD) assays (BioLegend, San Diego, CA # BLD‐640930), per manufacturer's guidelines as described (Goodis et al. 2024; Moses et al. 2021); IC_50_'s were generated in Prism's GraphPad by modeling a nonlinear fit (4‐parameter, variable Hill‐slope) against log(inhibitor) vs relative absorbance values; top and bottom constraints were set to 1 and 0, respectively. AnnV/7AAD assays were assessed by flow cytometry and analyzed using FlowLogic software v8.7.

## 3 Results and Discussion

**Figure 1:**
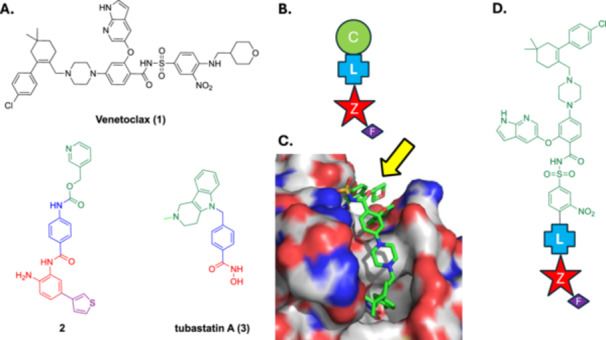
(A) Structures of the BCL‐2 inhibitor venetoclax (**1**), the HDAC1‐selective inhibitor **2**, and the HDAC6‐selective inhibitor tubastatin A (**3**); (B) Canonical pharmacophore of an HDAC inhibitor (HDACi): C, capping group; L, linker; Z, zinc‐binding group (ZBG); F (not always present, foot‐pocket‐targeting group; (C) Co‐crystal structure of venetoclax bound to BCL‐2 (PDB ID: 6o0p), with the solvent‐exposed tetrahydropyranyl group highlighted with a yellow arrow. (D) Rational design of BCL‐2/HDAC dual inhibitors, in which the bulk of venetoclax assumes the role of the capping group within the HDACi pharmacophore, and the linker, zinc‐binding group and foot‐pocket‐targeting group (if present) are grafted onto venetoclax in place of its solvent‐exposed (tetrahydro‐2*H*‐pyran‐4‐yl)methylamino motif.

Figure 1A depicts the structures of the BCL‐2 inhibitor venetoclax (**1**), HDAC1/2/3‐selective inhibitor **2** (Moradei et al. 2007), and the HDAC6‐selective inhibitor tubastatin A (**3**) (Butler et al. 2010). The canonical pharmacophoric model of an HDACi is provided in Figure 1B: a zinc‐binding group (ZBG), or “Z”, most typically a hydroxamic acid, chelates the zinc ion at the base of the active site; distal to the ZBG is a capping group, C, that sits on the protein surface; these components are connected together through a linker, L, that binds in the hydrophobic channel of the enzyme. Moreover, further functionality, F, may be grafted onto the ZBG to afford additional interactions within the foot‐pocket of the active site cavity (Melesina et al. 2021). Importantly, each of the capping, linker and ZBG modules may be tailored to enhance HDAC isozyme selectivities, for example the *ortho*‐aminoanilide ZBG in **2** imparts selectivity for HDACs 1‐3, while the phenyl hydroxamic acid of **3** promotes HDAC6‐selectivity (Melesina et al. 2021).

As mentioned earlier, favourable results have been reported in combination studies with HDACi's and venetoclax (Chen et al. 2009; Cryenne et al. 2017; Zha et al. 2023). In addition to Zhou and colleagues, (Zhou et al. 2019) we have demonstrated that the solvent‐exposed tetrahydropyranyl motif of venetoclax (Figure 1C) is an excellent position from which to graft supplementary inhibitors to generate polypharmacologic compounds, without impairing the ability to inhibit BCL‐2 (Goodis et al. 2024). Accordingly, towards boosting the anti‐leukemic activity of venetoclax, Figure 1D provides our general design strategy for dual BCL‐2/HDAC1 and dual BCL‐2/HDAC6 inhibitor candidates. Given that the molecular weight (MW) of venetoclax is 868 Da and that some of our dual inhibitors have MWs of around 1000 Da, we acknowledge that such large dual inhibitors may suffer from suboptimal cell penetration. As discussed previously, the HDAC1 and HDAC6 isoforms were chosen as the HDAC targets because HDAC1 and HDAC6 have been associated with the development and progression of CLL and AML, two leukemias for which venetoclax has already received FDA approval, and is currently undergoing investigation for the treatment of multiple myeloma and non‐Hodgkin's lymphoma, among other hematologic cancers (Gibson et al. 2021; Lasica and Anderson 2021). The structural determinants to accomplish selectivity for HDAC1 and HDAC6 are quite well understood (Melesina et al. 2021). Briefly, considering venetoclax as the capping group of an HDACi, our approach to prepare dual BCL‐2/HDAC inhibitors was to replace the (tetrahydro‐2*H*‐pyran‐4‐yl)methylamino motif of venetoclax with linkers L to which were attached zinc‐binding groups Z, with or without an associated foot‐pocket‐targeting group, F. Last, to achieve HDAC1‐ or HDAC6‐selectivity, initially the *ortho*‐aminoanilide ZBG in **2** (Moradei et al. 2007) or the phenyl hydroxamate ZBG in **3** (Butler et al. 2010) were deployed, respectively.

Accordingly, we initially designed and tested **BD‐4‐213** and **SF‐8‐038** as potential BCL‐2/HDAC1 and BCL‐2/HDAC6 dual inhibitors, respectively. Table 1 shows their structures and the relative inhibitory activities of HDAC1 and HDAC6, as quantified by HDAC1 and HDAC6 fluorogenic assays, wherein turnover (deacetylation) of an acetylated lysine‐containing substrate by these enzymes was monitored (see Experimental Section). **BD‐4‐213** was only a moderately potent inhibitor of HDAC1 (IC_50_ = 2.88 μM), but it pleasingly demonstrated > 10‐fold selectivity for HDAC1 over HDAC6, as predicted for this *ortho*‐aminoanilide ZBG (Melesina et al. 2021). Conversely, the phenyl hydroxamic acid derivative **SF‐8‐038** exhibited the predicted (Porter et al. 2017) potent inhibitory activity and selectivity (> 80‐fold) for HDAC6, with no inhibition of HDAC1 detected at up to 30 μM substrate concentration. Importantly, according to a BCL‐2 TR‐FRET assay, both **BD‐4‐213** and **SF‐8‐038** retained the potent (< 1 nM) inhibition of BCL‐2 exhibited by the parent drug venetoclax.

**Table 1:** IC_50_ HDAC1, HDAC6 and BCL‐2 inhibition data of dual BCL‐2/HDAC inhibitors.

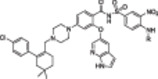							
Code name	R	HDAC1 (μM)	IC_50_		IC_50_ (nM)		
			HDAC6 (μM)	BCL‐2 (nM)	MV4;11	MV4;11 BCL‐2 OE	MOLM‐14
**BD‐4‐208**	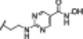	0.463 ± 0.060	0.149 ± 0.02	ND	.> 10,000	ND	ND
**SF‐8‐038**	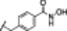	> 30	0.368 ± 0.085	0.617 ± 0.050	285 ± 81	2454 ± 96	> 10,000
**SF‐8‐083**	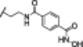	11.6 ± 1.76	0.418 ± 0.034	ND	.> 10,000	0.418	0.617
**SF‐8‐062**	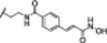	6.14 ± 1.24	0.314 ± 0.050	ND	.> 10,000	0.314	ND
**AMC‐4‐154**	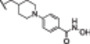	> 30	8.71 ± 1.89	0.554 ± 0.045	66.2 ± 13.6	631 ± 50	> 10,000
**BD‐4‐213**	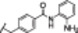	2.88 ± 0.57	> 30	0.539 ± 0.062	19.3 ± 5.6	342 ± 55	1630 ± 36
**AMC‐4‐074**	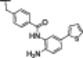	*Limited solubility*					
**AMC‐4‐075**	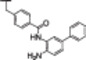	*Limited solubility*					
**AMC‐4‐101**	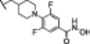	*Limited solubility*					
**Venetoclax**	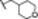	> 30	> 30	0.426 ± 0.049	82.82 ± 16.7	1021 ± 177	1718 ± 59
**Entinostat**	—	0.55	> 30	ND	ND	ND	ND
**Quisinostat**	—	0.987 ± 0.389	0.049 ± 0.012	ND	ND	ND	ND

[a] Venetoclax, quisinostat and etinostat are positive controls for the cell‐free BCL‐2, HDAC1 and HDAC6 assays, respectively. ND = not determined. Due to limited solubilities, data from testing **AMC‐4‐074**, **AMC‐4‐075** and **AMC‐4‐101** were not reliable, and thus not reported. Cell‐free assays were performed in duplicate, cell‐based assays at least in triplicate; errors are SEMs.

With these promising results in hand, we elected to expand our library of dual BCL‐2/HDAC inhibitors. First, replacement of the phenyl ring in the phenyl hydroxamic acid of **SF‐8‐038** with a pyrimidine, as in **BD‐4‐208**, retained potent inhibition of HDAC6 (IC_50_ = 0.149 μM), but resulted in significant reduction in HDAC6/1 selectivity to only ~3‐fold. Likewise, extension of the methylene linker to a CH_2_CH_2_NHCO linker (**SF‐8‐083**) resulted in an erosion in HDAC6/HDAC1 selectivity from > 80‐fold to ~30‐fold. Projection of the hydroxamic acid further from the phenyl ring, as in **SF‐8‐062**, yielded a potent HDAC6 inhibitor (IC_50_ = 0.314 μM) with about 20‐fold selectivity over HDAC1 (IC_50_ = 6.14 μM). Efforts to amplify the HDAC6/1 selectivity of **SF‐8‐038** by the introduction of one or two fluorine atoms meta to the hydroxamic acid functional group group (Sandrone et al. 2021) proved synthetically challenging. Instead, we prepared **AMC‐4‐154**, and its difluoro congener **AMC‐4‐101**, which we predicted would exhibit greater HDAC6/1 selectivity than **AMC‐4‐154** (Sandrone et al. 2021). Unfortunately, while **AMC‐4‐154** was > 3‐fold selective for HDAC6, its inhibition for this isozyme (IC_50_ = 8.71 μM) was reduced by more than an order or magnitude relative to parent compound **SF‐8‐038**. Furthermore, we were unable to confirm if **AMC‐4‐101** was more selective for HDAC6 due to its limited solubility under the assay conditions. Alongside this study, we attempted to enhance the HDAC1 inhibitory activity of **BD‐4‐213** through targeting the enzyme's foot‐pocket (Melesina et al. 2021; Moradei et al. 2007). We proposed to do this by grafting on a thiophene (**AMC‐4‐074**) or a phenyl ring (**AMC‐4‐075**) to the aniline ring of the *ortho*‐aminoanilide of **BD‐4‐213**, as guided by the ZBG in compound **2** (Figure 1A). Once again, solubility proved problematic, highlighting a common issue with polypharmacologic drugs based on venetoclax; these poorly soluble compounds were not evaluated.

Our dual inhibitors were next evaluated in an alamarBlue^TM^ cell metabolism/viability assay against 3 human AML cell lines: MOLM14, MV4;11, and MV4;11 BCL‐2 overexpressing (OE). As expected, venetoclax was a potent inhibitor of MV4;11 cells (IC_50_ = 82 nM), and was much less potent against the MV4;11 OE BCL‐2 cell line (IC_50_ = 1 μM). Venetoclax was only a moderately potent inhibitor of MOLM14 cells (IC_50_ = 1.7 μM). Several of our dual inhibitors were substantially less potent than venetoclax, which may be due to their larger sizes that may limit cell penetration and/or reduce solubilities under the assay conditions. However, pleasingly, several of our top compounds based on the cell‐free assay results also performed well in the AML cell‐based metabolism/viability assays. For example, **SF‐8‐038**, which was potent and HDAC6‐selective in our cell‐free assays, was a potent inhibitor of MV4;11 cell metabolism/viability, with an IC_50_ = 285 nM, only ~3‐fold less active than venetoclax, although SF‐8‐0938 was less active than venetoclax in the MV4; 11 BCL‐2 OE cells.

On the other hand, both the HDAC1‐selective **BD‐4‐213** and the HDAC6‐selective **AMC‐4‐154** were more potent against both MV4;11 and MV4;11 BCL‐2 OE cells than venetoclax. Since synergy was reported for the combination of venetoclax with the HDAC1/2/3‐selective inhibitor chidamide, (Gangping et al. 2021) the high activity of **BD‐4‐213** in the cell‐based assay may be due to the combined inhibition of both BCL‐2 and HDAC1 by this hybrid molecule. Meanwhile, the potent inhibition of cell metabolism/viability by **AMC‐4‐154** may be due to the combined inhibition of BCL‐2 and upregulation of the pro‐death BCL‐2 protein BIM, since inhibition of HDAC6 has been linked to the upregulation of BIM (Losson et al. 2020).

**Figure 2:**
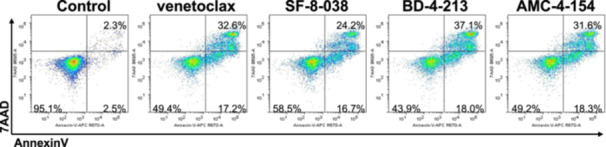
Apoptotic cell death of MV4;11 AML cells. Cells were incubated with 200 nM venetoclax, **SF‐8‐038**, **BD‐4‐213** or **AMC‐4‐154** in 48 h cultures before AnnV/7AAD staining. As a negative control, an equi‐volume of DMSO‐treated sample was also assessed. Flow cytometric dot plots of 200 nM‐treated MV4;11 cells stained with Annexin‐V and 7AAD depict the gating strategy used to delineate viable cells (AnnV‐/7AAD‐), early apoptotic cells (AnnV+/7AAD‐), and late apoptotic cells (AnnV+/7AAD +).

To verify that the observed drug‐induced reductions in cell metabolism/viability reflected in the results of alamarBlue assays were accompanied by apoptotic cell death, the established cellular mechanism of action of venetoclax (Souers et al. 2013), flow cytometric analyses of cell apoptosis/death were conducted for our 3 best venetoclax‐based dual BCL‐2/HDAC inhibitors **SF‐8‐038**, **BD‐4‐213** and **AMC‐5‐154** against MV4;11 cells (Figure 2). As a positive control, MV4;11 cells were treated with venetoclax (200 nM), which induced a potent (~50%) decrease in % AnnV‐/7AAD‐ (viable) cells, and essentially half of the cells had entered into early AnnV + /7AAD‐ (early) or AnnV + /7AAD+ (late) stages of apoptotic cell death. Treatment with 200 nM of either compound **BD‐4‐213** or **AMC‐4‐154** induced apoptosis similar to venetoclax, while **SF‐8‐038** was somewhat less potent, consistent with its relatively lower potency in the metabolism/viability assays (Table 1).

### 3.1 Chemistry

**Scheme 1:**
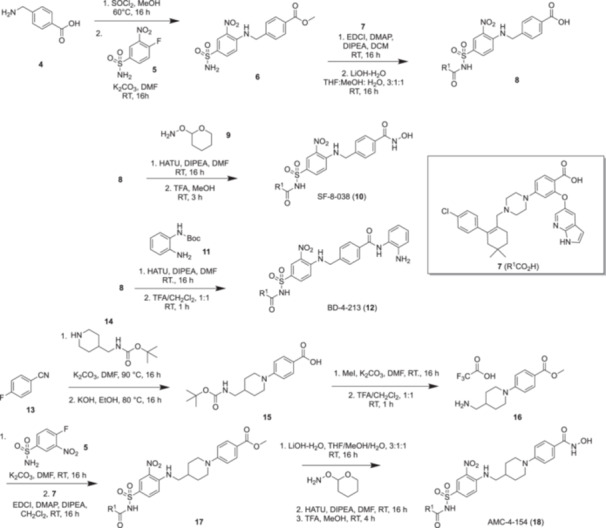
Syntheses of lead compounds SF‐8‐038, BD‐4‐213 and AMC‐4‐154.

**Syntheses of SF‐8‐038, BD‐4‐213:** Briefly, and as illustrated in Scheme 1, amino acid **4** was esterified with thionyl chloride in MeOH, then underwent a nucleophilic aromatic substitution (S_N_Ar) reaction with aryl fluoride **5** to yield **6**. Acylation of which with R^1^CO_2_H (**7**) followed by saponification of the methyl ester furnished acyl sulfonamide **8**. Next, the liberated carboxylic acid of **8** was coupled to *O*‐THP‐protected hydroxylamine (**9**), and gentle acidic hydrolysis of the THP protecting group was accomplished with stoichiometric TFA in MeOH, delivering the polypharmacologic BCL‐2/HDAC6 inhibitor **SF‐8‐038** (**10**). Alternatively, **8** was coupled to mono‐Boc‐protected *ortho*‐phenylenediamine (**11**), then removal of the Boc group furnished the polypharmacologic BCL‐2/HDAC1 inhibitor **BD‐4‐213** (**12**).

**Synthesis of AMC‐4‐154:** As depicted in Scheme 1, an S_N_Ar reaction between 4‐fluorobenzonitrile (**13**) and piperdine **14**, followed by hot basic hydrolysis of the nitrile yielded **15**. Esterification of the carboxylic acid in **15**, and then deprotection of the Boc group afforded amine **16**. Next, an S_N_Ar reaction of **16** with **5**, proceeded by an acylation with R^1^CO_2_H (**7**) delivered **17**. Once again, a sequence of saponification, coupling to **9** and deprotection yielded the polypharmacologic BCL‐2/HDAC6 inhibitor **AMC‐4‐154** (**18**).

The syntheses of all other target molecules are provided in the Methods section.

## 4 Conclusions

In summary, we successfully added motifs from established HDAC1‐selective and HDAC6‐selective inhibitors onto the clinical BCL‐2 inhibitor venetoclax, using several chemical tactics. From these, we identified hybrid molecules whose BCL‐2 inhibitory activities were not impaired, and whose HDAC6/1 selectivities were largely retained. Our most potent and selective dual BCL‐2/HDAC1 inhibitor **BD‐4‐213** and BCL‐2/HDAC6 inhibitor **AMC‐4‐154** inhibited the metabolism/viability of three AML cell lines more potently than did the parent BCL‐2 inhibitor venetoclax. All three lead dual BCL‐2/HDAC compounds (**BD‐4‐213**, **AMC‐4‐154** and **SF‐8‐038**) triggered AML cell apoptosis, the established mechanisms of activity of both venetoclax and HDACi's. Attempts to further enhance the potencies and selectivities of the dual BCL‐2/HDAC inhibitors proved difficult, owing to synthetic chemistry challenges and solubility issues. Future work should focus on the introduction of solubilizing functionalities into the linking groups, such as short polyethyleneglycol (PEG) units or titratable secondary or tertiary amines, to counter the higher molecular weights.

## Data Availability

The data that supports the findings of this study are available in the Supporting information of this article.
